# BREATHLEssness in INDIA (BREATHE-INDIA): realist review to develop explanatory programme theory about breathlessness self-management in India

**DOI:** 10.1038/s41533-025-00420-2

**Published:** 2025-03-13

**Authors:** Joseph Clark, Naveen Salins, Mithili Sherigar, Siân Williams, Mark Pearson, Seema Rajesh Rao, Anna Spathis, Rajani Bhat, David C. Currow, Kirsty Fraser, Srinagesh Simha, Miriam J. Johnson

**Affiliations:** 1 Wolfson Palliative Care Research Centre, University of Hull, Hull, UK; 2 Department of Palliative Medicine and Supportive Care, Kasturba Medical College Manipal, Manipal Academy of Higher Education, Manipal, India; 3 Bangalore Hospice Trust - Karunashraya Institute for Palliative Care Education and Research, Bengaluru, India; 4 International Primary Care Respiratory Group, London, UK; 5 University of Cambridge, Cambridge, UK; 6 SPARSH Hospital, Bengaluru, India; 7 IMPACCT (Improving Palliative, Aged and Chronic Care through Clinical Research and Translation), Faculty of Health, University of Technology Sydney, Sydney, NSW Australia; 8 Bangalore Hospice Trust - Karunashraya, Bengaluru, India

**Keywords:** Respiratory signs and symptoms, Health care

## Abstract

Breathlessness is highly prevalent in low and middle-income countries (LMICs). Low-cost, non-drug, breathlessness self-management interventions are effective in high-income countries. However, health beliefs influence acceptability and have not been explored in LMIC settings. Review with stakeholder engagement to co-develop explanatory programme theories for whom, if, and how breathlessness self-management might work in community settings in India. Iterative and systematic searches identified peer-reviewed articles, policy and media, and expert-identified sources. Data were extracted in terms of contribution to theory (high, medium, low), and theories developed with stakeholder groups (doctors, nurses and allied professionals, people with lived experiences, lay health workers) and an International Steering Group (RAMESES guidelines (PROSPERO42022375768)). One hundred and four data sources and 11 stakeholder workshops produced 8 initial programme theories and 3 consolidated programme theories. (1) Context: breathlessness is common due to illness, environment, and lifestyle. Cultural beliefs shape misunderstandings about breathlessness; hereditary, part of aging, linked to asthma. It is stigmatised and poorly understood as a treatable issue. People often use rest, incense, or tea, while avoiding physical activity due to fear of worsening breathlessness. Trusted voices, such as healthcare workers and community members, can help address misconceptions with clear, simple messages. (2) Breathlessness intervention applicability: nonpharmacological interventions can work across different contexts when they address unhelpful beliefs and behaviours. Introducing concepts like “too much rest leads to deconditioning” aligns with cultural norms while promoting beneficial behavioural changes, such as gradual physical activity. Acknowledging breathlessness as a medical issue is key to improving patient and family well-being. (3) Implementation: community-based healthcare workers are trusted but need simple, low-cost resources/skills integrated into existing training. Education should focus on managing acute episodes and daily breathlessness, reducing fear, and encouraging behavioural change. Evidence-based tools are vital to gain support from policymakers and expand implementation. Breathlessness management in India must integrate symptom management alongside public health and disease treatment strategies. Self-management interventions can be implemented in an LMIC setting. However, our novel methods indicate that understanding the context for implementation is essential so that unhelpful health beliefs can be addressed at the point of intervention delivery.

## Background

Breathlessness, a common symptom associated with long-term illness often persists despite the underlying causes being optimally manged, leading to what is known as chronic breathlessness syndrome^1^. It contributes to physical and psychological distress of both individuals and their families. The increasing burden of diseases that cause chronic breathlessness is higher in Low and Middle-Income Countries (LMICs). However, most research on breathlessness has been conducted in High-Income Countries (HICs), resulting in a notable evidence gap concerning the effectiveness of breathlessness interventions when implemented within the diverse health and social systems, cultural beliefs, and characteristics of LMICs settings. One study conducted in Sudan and Tanzania reports difficulties of physical work in context of respiratory problems, extreme heat and dusty environment^2^. However, no study directly addresses the impact of breathlessness in an LMIC.

Evidence from high-income countries (HICs) suggests that breathlessness leads to a detrimental cycle of physical disability, reduced physical activity^3^, deconditioning, anxiety, and worsening breathlessness^4^. It impacts individuals’ daily functioning, ability to work (absenteeism and presenteeism), emotional well-being, and family dynamics. Over 50% of individuals experiencing breathlessness experience anxiety, either as a cause or a consequence of their condition^5^. Furthermore, breathlessness is associated with poorer survival rates, diminished health-related quality of life, and elevated disability scores across all adult age groups^6^. Affected individuals tend to contribute less to the workforce^7^, leading to lower household incomes and high caregiving costs, heightening the risk of poverty^8^ and forcing families to compromise their children’s education to supplement income or provide informal care^9^. There is evidence from LMICs that the impact of breathlessness mirrors closely HICs. However, these issues may be even more widespread and damaging in LMICs, where activities of daily living are commonly in context of high rates of poverty and greater exposure to environmental factors (e.g. air pollution, heat, cold)^10^.

Best available evidence for managing the symptom of breathlessness is for non-pharmacological interventions like breathing techniques^11–15^, body positioning^16–19^ facial cooling^20–23^, anxiety management^24^, and paced physical activity^14^. These interventions require little clinical knowledge to deliver, and are teachable to patients, lay workers, family caregivers and clinical staff. Importantly, these interventions are low cost at the point of delivery and can be deployed in community settings. However, complex interventions developed in HICs are likely to fail in LMICs unless adapted to local contexts^25^.

We conducted a realist review^26–28^ with stakeholder involvement, to develop theory and co-design a breathlessness intervention for use in India. Breathlessness is common in India, but symptom focussed interventions are not widely available. A culturally heterogeneous population close to 1.5 billion people means there is likely be a complex range of cultural norms, contexts and values likely to influence health beliefs and behaviours about breathlessness. Our co-design work will be presented separately, but here we report our realist review which aimed to;

- Understand how breathlessness self-management works in “real-life” population and individual contexts;
- Understand contexts (e.g., country, setting, community systems, beliefs, intervention components) for effective implementation; and
- Develop explanatory programme theory about breathlessness self-management in India.

## Design

We conducted a realist review^25^ drawing on RAMESES publication standards^29^ and registered the protocol on PROSPERO [CRD42022375768]^30^. The iterative and flexible approach enabled by a realist review, together with its focus on identifying context-sensitive explanatory mechanisms that enable the development of theory^31^ meant that realist review was well-suited to exploring the complexity of breathlessness management in India. We draw on realist philosophy which allows exploration of ‘generative mechanisms’ relevant to breathlessness and breathlessness management^32^. Our project team comprised; a Project Management Group, a Steering Group and three Stakeholder Groups: (1) doctors; (2) nurses and allied health professionals; and (3) people with lived experiences of breathlessness (including carers). Members of our Project Management Group nominated and invited Stakeholders to participate and Experts to join our Steering Group. Stakeholders subsequently invited other Stakeholders to join groups. Stakeholder workshops took place online, using Microsoft Teams facilitated by a member of the Project Management Group. Groups met separately to avoid power imbalances and ensure all views were heard. Two researchers (JC and MN) were present for all meetings and took notes.

## Methods

**Fig. 1:**
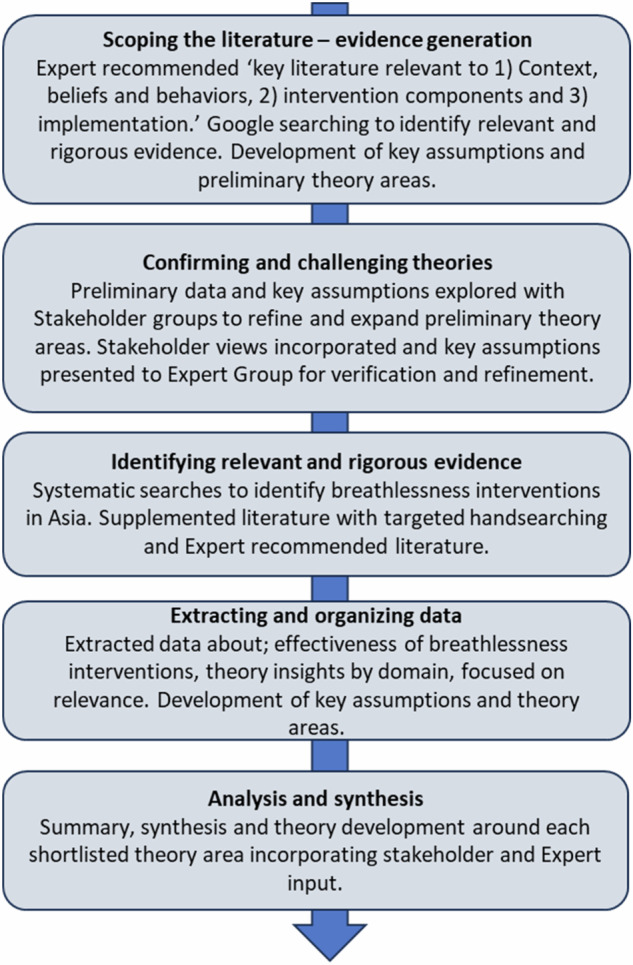
Stages of our realist review.

Key stages of the review are presented in Fig. 1. and outlined beneath. Ethical approval including for waiver of consent from stakeholders was obtained from Karunashraya Ethics Group (India) and Hull York Medical School Ethics Committee (REF: 22-23 33, United Kingdom).

### Scoping the literature - evidence generation

We initiated this project by seeking recommendations from our Project Management and Steering Group for key literature and data sources related to managing breathlessness and healthcare delivery in low-resource settings. Our focus encompassed any data source pertinent to beliefs and behaviours surrounding breathlessness, interventions designed for breathlessness, factors that may influence the acceptability of these interventions, and healthcare delivery, emphasising Asia. We did not assess quality of included sources, our focus was on relevance to theory generation^33^. Given the limited literature directly addressing breathlessness in India, we identified relevant data for presentation to and interpretation by our stakeholders and Steering Group. We also conducted iterative searches using Google to explore emerging evidence from the literature recommended by experts.

### Confirming and challenging emerging theories – Stakeholder Involvement

We extracted key insights from recommended data sources and developed ‘if, then’ statements to support development of preliminary theory areas and assumptions^28^. Statements were organized into three categories: (1) context, beliefs and behaviours; (2) intervention; and (3) implementation, and sent to all members of the Project Management Group for comment. Comments were discussed at Project Management Group meetings. ‘If, then’ statements were consolidated and presented to Stakeholder Groups.

**Fig. 2:**
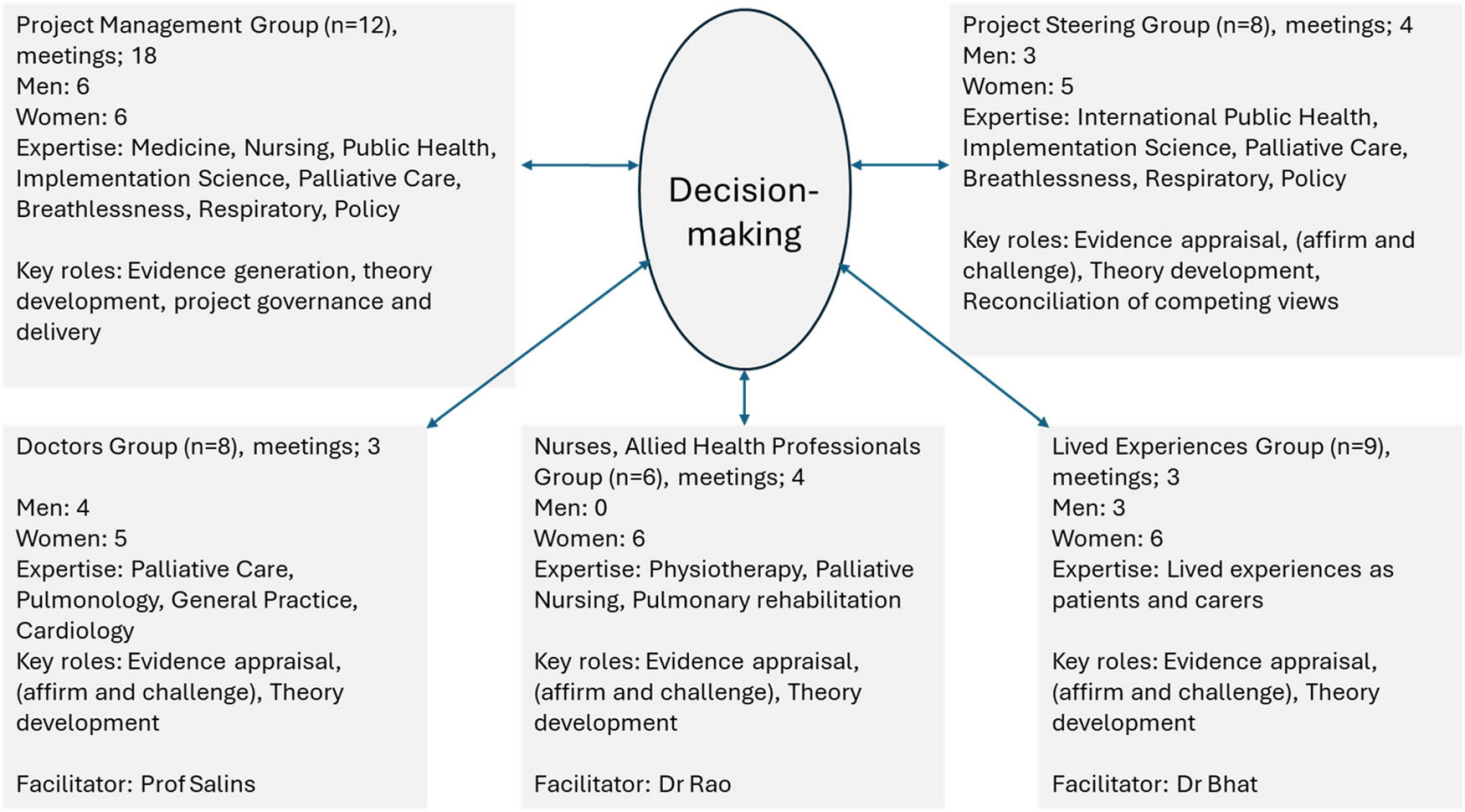
Stakeholder Involvement and Governance.

We held three ‘rounds’ of meetings with Stakeholder Groups (*n* = 10) and our Steering Group (n = 4) [Fig. 2].

**Fig. 3:**
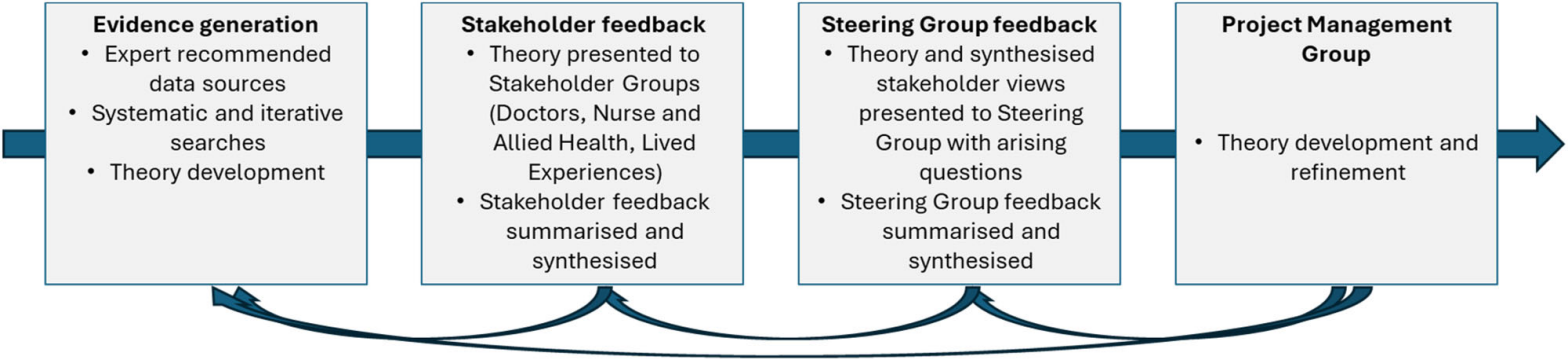
Theory development process.

Materials were sent to Stakeholders prior to each meeting for review. Stakeholders were then asked for their views on the data we generated regarding explanatory value, credibility and gaps. Stakeholder views were used to refine consolidated statements. Consolidated statements with Stakeholder views were presented to our Steering Group for discussion and conceptual clarity of emergent theory. Our Process of theory generation is presented in Fig. 3.

### Identifying relevant evidence

We used a systematic review of breathing exercises in people with COPD as a starting point for systematic searches^34^. We then conducted a systematic search of one database using MEdical Subject Headings (MeSH) terms (dyspnoea, breath*, intervention, self-management), restricted to Adults, post-2000, conducted in Asia and not restricted by study design [Supplementary File 1]. Two reviewers (KF, MN) screened articles for inclusion, a third (JC) adjudicated disagreements. In our Protocol we planned to search at least five databases. However, because of the richness of data generated from one database, the Steering Group decided that additional database searches were unnecessary.

### Analysis and synthesis

All data sources were coded and summarised; how they were identified (expert recommended, iterative searching, systematic search), contribution to our synthesis (high, medium, low) and domain (Context Beliefs and Behaviours, Intervention Development, Intervention Delivery and Implementation)^35^. We identified important Contexts, Mechanisms and Outcomes (CMO) and developed consolidated CMO statements. The Project Management Group offered critique of the development of programme theories and refinements were made. All members agreed the final synthesis.

## Results

**Fig. 4:**
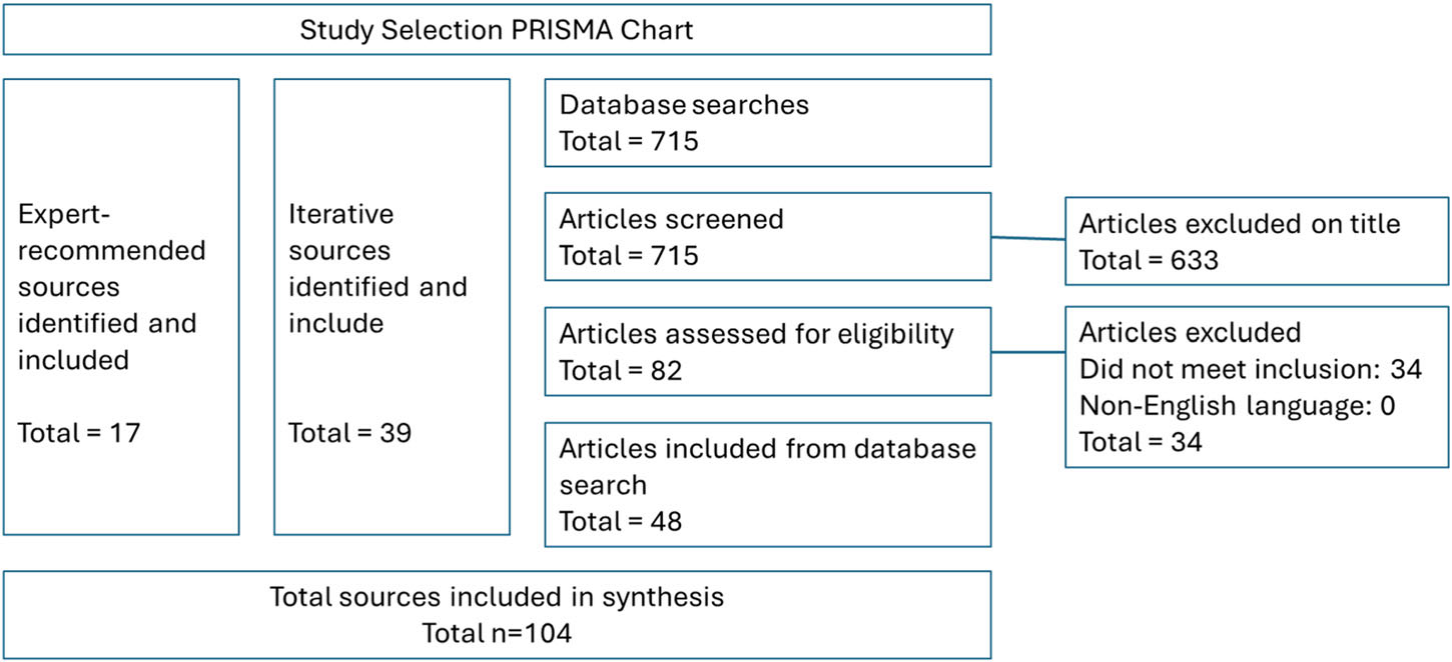
Study selection.

We included 104 sources: 17 recommended by members of our Project Management Group or Steering Group; 48 identified by systematic review and 39 identified iteratively. A summary of included sources is presented in Supplementary File 2. Our study selection process is summarised in Fig. 4.

We developed eight initial CMO configurations. Key results and initial programme theories are presented in Table 1. CMO configurations were refined using the located sources. enabling the development of final context-specific intervention and implementation programme theories [Box 1]. Published sources are cited and stakeholder views are indicated by group.

**Table 1:** Key results and Initial Context-Mechanism-Outcome configurations, presented as ‘If, Then’ statements.

	Key findings	If, then statement
1. Identifying the breathless population	• Forty four percent of adults in India report breathlessness limiting exertion • Breathlessness may have multiple (un)diagnosed causes. • Non-medical factors (e.g. poverty, malnutrition, post-tb) are important drivers of breathlessness prevalence	**If** breathlessness self-management approaches are to be encouraged safely, **then** ‘red flags’ must also be taught to promote seeking additional clinical support when relevant.
2. Health-seeking – search for a cause and a cure	• People search for cure for underlying cause and pay costly fees, often to multiple providers • Breathlessness often perceived as ‘normal,’ as part of illness or ageing • Health decisions commonly made at family-level	Symptom management must be delivered alongside reversal of modifiable causes and treatment of medical cause. **If** people are to engage with symptom management, **then** they must not feel that addressing symptoms is at the expense of modifying therapies and reversal of modifiable causes.
3. Health workforce	• Most clinicians unsure how to treat the symptom of breathlessness (therapeutic helplessness) • Medicines are prescribed for undiagnosed cause(s) • Poor coordination of care, history-taking	Witnessing breathlessness is distressing to health workers. **If** health workers are trained to teach breathlessness self-management, **then** they will apply their training because it also addresses their own unmet need.
4. Fatalistic and passive avoidance	• People with breathlessness ‘do less’ over time and lose fitness • Become accustomed to breathlessness as ‘inevitable and don’t seek help • Breathlessness stigmatised in communities	Understanding that breathlessness is not inevitable is key to changing behaviours. **If** patients/families can be taught the relationship between breathing, thinking and functioning, **then** they are more likely to engage with self-efficacy approaches.
5. Active avoidance	• People with breathlessness avoid activities because breathlessness is frightening	Addressing fear is key to promoting lifestyle changes. **If** self-management approaches can help people feel safe, **then** they are more likely to understand breathlessness as a healthy bodily response and not something to be avoided.
6. Self-management	• 41 interventional studies identified by systematic searches show neutral or positive effects on breathlessness outcomes • Non-pharmacological interventions more acceptable in community settings • Experiencing benefits is key to improving outcomes	Any breathlessness-targeted intervention may be helpful, because acknowledgement – or education - of breathlessness as a clinical problem is itself helpful to people living with breathlessness. **If** people find self-management approaches helpful **then** they will continue to use them.
7. Health beliefs	• People trust modern medicine but also trust their own health beliefs • Ayurvedic understandings of health underpin beliefs (e.g. food is an important aspect of health) • Framing of breathlessness self-management as modern medicine is key to engaging families and improving patient outcomes	. **If** self-management education is to gain credibility, **then** it must be kept simple and address patient/family concerns at the point of delivery.
8. Care in the community – place influences expectations	• Non-pharmacological interventions are consistent with pexpectations when delivered in the community (people don’t expect medicine) • Community health workers have a high workload, but are ideally placed to receive and administer breathlessness education	Delivery of non-pharmacological healthcare in the community is consistent with expectations. **If** breathlessness self-management is brought into the community, **then** communities will support implementation because breathlessness will be normalised as a modifiable problem and address unmet needs in communities.

Box 1 Programme Theories ***Context***

Breathlessness is common in communities, may have multiple causes (illness, lifestyle, malnutrition, environment). People’s experience and understanding of breathlessness is primarily framed by local beliefs about illness and healthcare (ayurvedic understandings, health problems need medicine) rather than a (Western) medical diagnosis and treatment plan. Breathlessness is stigmatized in communities and often misattributed to asthma or other causes. It is also commonly misunderstood as a natural part of ageing or as a hereditary issue rather than a modifiable problem.

Persistent breathlessness is frightening to the person and their support network, who develop coping mechanisms (e.g. incense, aromatherapy, oxygen, massage, tea). Physical activity is avoided by individuals who may perceive ‘exercise’ as ‘sport’ and is discouraged by family members to avoid breathlessness. Language is needed which reflects the pervasiveness of breathlessness in the lives of individuals and family members, its psychological and physical impact.

Despite unhelpful beliefs and behaviours, when accessing healthcare people want and expect to be ‘told what to do’ (*dos and don’ts*) from a trusted person who offers them hope. Healthcare workers are ‘trusted voices’, but so are members of their communities; both these groups may share unhelpful beliefs and behaviours. In the context of limited awareness of the causes of breathlessness, and the relationship between ‘breathing, thinking and functioning’, resources must be developed which focus upon cognitive change and ‘messages saying what to do’ must be kept simple.

***Intervention***

Interventions are needed that address breathlessness of unknown, or multiple cause, whilst promoting appropriate health-seeking. Breathlessness interventions which works in one context, are likely to work in another, where unhelpful beliefs and behaviours of individuals and families, can be anticipated and addressed as part of delivery of a breathlessness management intervention. Any non-pharmacological intervention may work (in any setting and for any disease group) because acknowledgement of breathlessness as a legitimate medical problem is fundamental to improving patient and family wellbeing.

• e.g. Normative responses to illness are to ‘take rest’ or reduce activity, with consequent deconditioning. Introducing the idea that a person with illness can take ‘too much rest’ engages with normative behaviours whilst focussing upon behavioural change (promoting activity).

***Implementation***

Community and primary healthcare workers are overworked and underpaid, but still the best placed groups to deliver a breathlessness intervention as trusted and visible members of communities. Educational resources should be low-cost at the point of delivery, kept simple and developed to be incorporated into existing training programmes of healthcare workers. Education of healthcare workers and people with breathlessness (plus families) should focus on cognitive change, breathlessness ‘essentials.’ Healthcare workers should be made aware of contextual factors to address during intervention delivery. Evidence supporting educational resources is necessary in order to gain influence with regulatory and government agencies.

There is a conceptual distinction between self-management approaches which aim to promote; (1) ability to manage episodes of worse breathlessness (2) ability to manage the everyday living with breathlessness – a daily practice of managing the inevitable increases in breathlessness that occur during physical and emotional activity. Episodes of breathlessness promote fear. If self-management approaches can help people feel ‘safe’ then they become more likely to adopt behavioural changes to improve everyday living with breathlessness.

### Identifying the breathless population

A cross-sectional study surveying 3000 community-dwelling adults in India found that 44% reported breathlessness limiting their exertion (mMRC ≥1)^36^. This indicates that breathlessness in communities across India is significantly higher than HICs. Moreover, this phenomenon is pronounced due to additional risk factors in India and varying access to and delivery of healthcare services (Doctor Group). Our initial focus was to investigate breathlessness within the context of respiratory illnesses, especially considering India’s high rates of chronic obstructive pulmonary diseases (COPD)^37,38^. However, all Stakeholder groups rejected this narrow focus, emphasizing the importance of understanding breathlessness throughout life and concerning psychological issues, obesity, and health conditions less frequently seen in HICs, including anaemia, post-tuberculosis effects, and malnutrition^39^. Amidst a variety of clinical, environmental, lifestyle, workplace, and poverty-related factors that can interact to cause breathlessness, respondents commonly attributed their symptoms to poor nutrition (28%), lung conditions excluding tuberculosis (17%), or anaemia (13%). Notably in the national survey, 12% of respondents were unsure about the cause of their breathlessness.

Health literacy is generally low in India, with many people rarely being able to access professional health services^40^. Existing definitions of chronic breathlessness often assume that the underlying cause has been identified and optimally managed. However, members of the Doctor Group, and the Nurses, and Allied Health Group, felt it could not be assumed that the underlying causes of breathlessness had been addressed appropriately.

### Health-seeking – search for a cause and a cure

Where breathlessness is identified as indicative of a health problem with a modifiable cause, people with breathlessness seek healthcare aimed at addressing reversible causes^41,42^. Diagnosing a cause of breathless can be challenging – especially when breathlessness may have multiple causes. Stakeholders with lived experiences confirmed difficult and expensive processes of accessing multiple providers in search for a diagnosis. Whether diagnosed or not, health professionals and communities often view breathlessness as a normal part of ageing or a result of unspecified biological changes (Doctor Group and Lived Experience Group)^39^.

Individual health behaviours in India are significantly influenced by family dynamics, which can either encourage or discourage health-seeking behaviour. Families often play a crucial role in interpreting clinical advice. Patients and their relatives may undertake long journeys and incur substantial costs to consult a preferred healthcare provider^34^. Rural populations are particularly disadvantaged. High costs are frequently (mis)interpreted as quality indicators quality (Doctor Group). Out-of-pocket spending can often lead to household poverty. When clinical treatments prove ineffective, families continuously negotiate decisions regarding healthcare access and provider choice, considering available resources. Stakeholders reported instances of ‘California daughter syndrome,’ where family members living overseas influence health recommendations based on services available to them—even if not applicable to context of India (e.g., “Can’t you find a better doctor?” (Doctor Group)). Women in particular, may face disadvantages in resource allocation, especially when residing in their husband’s family home, which is a common practice (Nurse and Allied Health Group).

### Health workforce

If people seek help for the *symptom* of breathlessness, they are likely to be met with a health workforce which is not trained to identify nor manage breathlessness. People accessing primary care services may receive an appointment of barely two minutes long^43^. This means that there is no time for ‘history taking’ to identify causes that could be addressed and people are triaged with minimal information. Use of multiple health providers and a fragmented health systems means that health workers have little relational continuity with their patients and medical notes are not shared between settings (Doctor Group and Steering Group). Some people maintain regular contact with a ‘family doctor’ although this is becoming less common. Where people *do* have a family doctor, they use them to arbitrate medical advice given in other healthcare settings. Many people access traditional healers, either as a primary or adjunct to seeking medical help.

Health workers want to help and commonly prescribe medicines (e.g. inhalers, antibiotics) – even if there is no diagnosis of reversible airways disease or identified infection^8^. This approach is consistent with medical training to identify and treat reversible causes and meets patient expectations for their healthcare. Despite perceptions that western medicines ‘overmedicate,’ when accessing healthcare, patients and their families expect to be prescribed medicines for health problems^44^. Most symptom management is provided in palliative care services, available to only a small percentage of the population, and access is sporadic^45^. Pulmonary rehabilitation also includes symptom-focussed interventions, although again, only a fraction of need is met; predominantly for people living with COPD. Doctors without specialist palliative care training report ‘therapeutic helplessness’ faced with patients with breathlessness^46^. Like family members, health workers are distressed when seeing people with acute-on-chronic breathlessness, without the skills to provide support.

### Fatalistic and passive avoidance

The Breathing-Thinking-Functioning educational tool conceptualises the cyclical relationships between breathlessness, anxiety and deconditioning. Breathlessness causes (and is caused by) distress, physical activity reduces to avoid becoming breathless which in turn deconditions the body, making breathlessness more likely. Stakeholders confirmed that this relationship is relevant in the context of India, with high levels of contextual complexity, passive – and active avoidance behaviours.

Many people with breathlessness do not access healthcare, either due to inaccessibility of services, or because of fatalistic attitudes to breathlessness. Absence of health-seeking does not mean that people are unaffected by breathlessness (Doctor Group, Nurse and Allied Health Group and Steering Group). (Mis)perceptions of the causes of breathlessness (e.g. occupation, previous infection of family history, old age) promote fatalistic attitudes towards its presence^24^. Fatalism discourages engagement in behavioural approaches aimed at improving coping and resilience to breathlessness^47^. It can also be misunderstood as an avoidance behaviour. However in passive avoidance, rather than avoid activities, people simply do less over time and lose fitness (Steering Group).

Breathlessness may be stigmatised in communities. Misperceptions of the causes of breathlessness isolate individuals, due to perceived risk of infection – especially in the context of cough (Lived Experiences Group). The COVID-19 pandemic reinforced perceptions and stigma. Stakeholders reported how whole families could be stigmatised, as ‘breathless families’ where multigenerational households are exposed to the same risk factors and experience similar health states. Stigma provokes people to undertake ‘the sick role’ in which they do less, are seen less in communities and worsen their health through deconditioning^42^. Stigma of breathlessness may be additional to biases against people with psychological illness^48^.

Despite stigma, breathlessness-targeted interventions delivered in the community can be beneficial to people with breathlessness and their families. Group activities like singing groups for people with COPD, promote relaxation, are associated with reduced psychological illness and improve quality of life^49,50^.

### Active avoidance

Active avoidance is not an incidental behaviour, but deliberate limitation of activity driven by fear of breathlessness and its consequences. Exploring the issue of breathlessness with stakeholder groups, attention immediately focused on worst, frightening breathlessness – particularly for people with lived. People with breathlessness describe frightening sensory experiences^51^ and fear that they may die because they ‘cannot get enough air’ (Lived Experiences Group). Distress *spreads through the family* as family members watch on, without the skills or knowledge to support their loved one (all Stakeholder Groups).

Households with a person with breathlessness face ongoing challenges in the context of air pollution, heat, cold – and especially where there is use of oxygen therapy. Oxygen is increasingly available and used in India as symptom control for breathlessness – although oxygen provides no additional benefit than cool airflow in those who do not qualify for long-term-oxygen therapy, or who do not desaturate on exertion^52^. Stakeholders with experience of caring for a person with breathlessness, describe anxiety relating to; constant monitoring of oxygen saturation levels, fears oxygen will run out and fears of oxygen as an explosive material (Lived Experience Group). Individuals and family members can become fixated on having oxygen available, something which itself shrinks social worlds, as people become unwilling to stray far from home.

### Self-management

A range of interventional studies conducted in Asia demonstrate positive effects for non-pharmacological interventions such as pulmonary rehabilitation^53^, music therapy^54^, yoga^55^, and pursed-lip breathing^56^. Among the 41 interventional studies identified by our systematic searches nearly all show neutral or positive effects on breathlessness outcomes. Non-pharmacological interventions are generally well-accepted in settings where medication is not expected to address health concerns, such as in community environments. Experiencing the benefits of self-management approaches is essential for improving health outcomes (Nurse and Allied Health Group). However, individuals may resist interventions designed to help them increase their activity levels (Nurse and Allied Health Group). Many perceive exercise only as ‘sport.’ Those who actively avoid activity often need support and guidance on what they can do, especially in managing their fear of breathlessness. Nonetheless, poverty serves as a significant barrier to effective self-care. Stakeholders with lived experience have shared various strategies to calm themselves and their loved ones, including listening to music, practising massage, and using aromatherapy oils.

### Health beliefs

Patients and their families trust their doctors, although trust is constantly reviewed, and people also hold to their own health beliefs. Ayurveda is commonly practiced in India and lay health beliefs associate health and illness with - principles of hot and cold^57^. Food is an essential component of good (or bad) health. Different types of food are perceived as beneficial (e.g. ginger), or harmful (e.g., foods inducing cold and phlegm like milk). Doctors report that their patients lose confidence in their advice if it does not acknowledge food as an important component (Doctor Group). There are concerning reports of people being denied food for fear that the act of eating will make them breathless (Nurse and Allied Health). A literature review of pulmonary rehabilitation for low-resource countries, addresses the important issue of food, emphasising the need for good nutrition, to be achieved by smaller meals more regularly^45^.

Allied Health stakeholders reported experiences where patients and families were resistant to interventions such as the handheld fan and again linked this to Ayurvedic understandings of health. Cold weather is perceived to worsen breathlessness amongst lay people and health professionals (All Groups). Strategies to keep the patient warm are reported as problematic; socks make the patient feel ‘restricted’ and heavy blankets are ‘suffocating’ (Lived Experience Group). Involving the family, or support system, in interventions can improve patient and family outcomes^48^. However, without appropriate education, family members may unintentionally reinforce negative health behaviours. For example, although it is good for a person to do activities which make them breathless to maintain strength and continue their usual activities, family members often encourage avoidance behaviours because it can be distressing to see their loved one breathless (All Groups).

### Care in the community – place influences expectations

Place of care influences patient expectations. In medical settings, people expect to be prescribed medicines, but there is no expectation of pharmacological solutions when care is delivered in the community (Nurse and Allied Health Group). Task shifting – asking non-physician healthcare workers to perform tasks traditionally undertaken by physicians – is a promising method for improving access to non-urgent healthcare in LMICs^58^.

A range of community-health workers operate in India, (e.g. Anganwadi Workers and Accredited Social Health Activists (ASHA). Both groups are reported to be responsive to training regarding identification and management of psychological illness and able to challenge stigma in communities^59^. Stakeholders agreed that despite a high workload and concerns over renumeration, ASHA are best-placed to receive and deliver a breathlessness intervention (Doctor Group and Nurse and Allied Health Group). ASHA are community health worker employed by the Ministry of Health and Family Welfare (MoHFW). ASHA are selected from the communities which they serve and have a structured education program which aims to equip them with the skills to identify and manage simple health problems, triage and promote health in communities. Despite having low levels of education, ASHA provide essential services and are trusted members of communities^60,61^. They may also share unhelpful health beliefs (Doctor Group and Nurse and Allied Health Group). Any intervention promoting breathlessness education to e.g., ASHA workers must be kept simple, challenge misperceptions and focus on cognitive change (Steering Group).

## Discussion

**Fig. 5:**
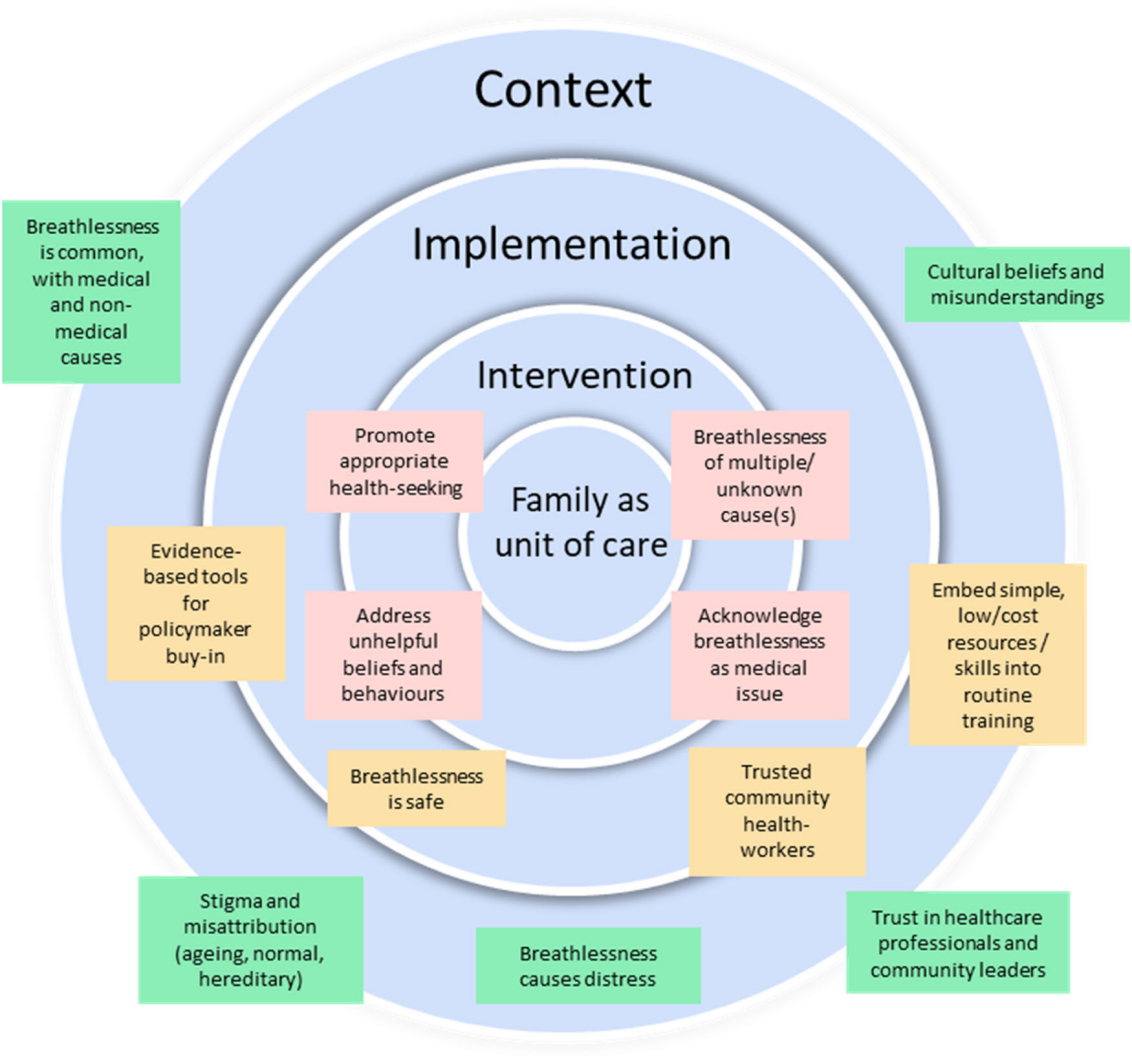
Final programme theories.

Breathlessness is common in India, may have multiple unaddressed causes and has widespread negative impact on families^29^. A summary of final programme theories is presented in Fig. 5.

Our review has identified how research developed in high-income settings has explanatory value for breathlessness in India (e.g. relationship between breathing, thinking, functioning). However, we have also identified additional contexts and challenges in India that are new to breathlessness research. A complex range of health beliefs and behaviours influence health-seeking behaviour, the healthcare which is offered and acceptability of symptom-targeted interventions. People with breathlessness may or may not seek healthcare for the symptom. Those who do, may expect a pharmacological treatment and are likely to receive one from a healthcare workforce which is not accustomed to treating symptoms or using self-efficacy approaches. Those who do not seek healthcare are also likely to suffer negative effects of breathlessness, but are unlikely to be known to health services. People with breathlessness *and* health workers use trusted strategies to address the problem, which may be ineffective. Acknowledgement of breathlessness as a therapeutic target, not something which is inevitable, is crucial for improving outcomes. Our finding that breathlessness may be of unknown cause and that optimal management of underlying causes is unlikely to be a context for long-term breathlessness, highlights a need for new approaches.

Our findings are consistent with a systematic literature review focussed on studies published in high-income countries developed the concept of ‘breathing space.’ Breathing space highlights how engaged coping and appropriate help-seeking (patient) and attention to symptom (clinician) helps maximise the patient’s quality of living with breathlessness^47^. In India, we identified how Health workers, people with breathlessness and their family members *actively* engage with breathlessness, but in ways which are unlikely to provide symptomatic relief. We identified fatalism, which resonates with breathing space’s concept of disengaged coping, but also highly engaged people with breathlessness and their families, applying their own health beliefs, often in the absence of professional support.

### Implications for practice

Breathlessness is a symptom which can be used as part of diagnostic pathways, *and* a symptom which impacts negatively on individuals and families *despite* optimal management of the cause. Wherever health workers encounter people with breathlessness, optimal management of underlying causes should not be assumed. Reversible causes may also be present and should be identified by detailed history-taking. However, symptom management should not wait for optimisation of medical treatments.

Breathlessness self-management approaches are already in use in India, primarily in palliative care services and in context of COPD management, however, coverage is limited. Extending symptom management requires that breathlessness education is provided at additional levels of healthcare. Many health workers *and* patients and families are conditioned through education and experience, that health problems are likely to have pharmacological solutions, or that they are a natural part of the life course. Understanding that breathlessness is not inevitable and, even if it is an expected symptom, can be helped, either as part of illness-focussed treatment, lifestyle interventions or even when no solution to the underlying cause is possible, is paramount. An educational intervention which targets, health professionals *and* people with lived experiences is needed.

Breathlessness self-management interventions are likely to have the same biological mechanisms of action in LMICs as in HICs. However, adaptation of how interventions are ‘framed’ is necessary at the point of delivery to address health beliefs of behaviours of individuals and their families. A ‘teach the teacher’ approach is one which empowers trainers to adapt their teaching at the point of delivery and respond to feedback in order to refine teaching in a two-way process. This allows teachers to address contextual issues, whilst delivering key educational information^62^. Self-management interventions are more likely to be helpful and used if unhelpful beliefs are acknowledged at the point of healthcare delivery.

Promoting increased, or maintenance of physical activity is an essential aspect of breathlessness care. Yoga, Tai Chi and Xi Gong are all exercise-based practices which positively influence breathlessness outcomes. However, we identified beliefs and behaviours which perceive exercise as something to be avoided in context of breathlessness. Improving community awareness of the safety and helpfulness of breathing-based cultural practices in context of breathlessness is a promising approach to empowering communities to participate in their own healthcare in a sustainable manner.

### Implications for research

Evidence of effectiveness of breathlessness self-management interventions developed in HICs appears relevant to the context of India. However, implementation research is needed to confirm that effectiveness is not lost when delivered in low-resource contexts and to develop an evidence-base for sustainable implementation. Effectiveness-implementation hybrid designs are needed to develop an evidence-base for breathlessness self-management interventions in India – and other LMICs^63^.

As well as evaluation of existing interventions, further intervention development is needed in context of local beliefs and behaviour. The NIHR-funded RECHARGE programme of work is one example of researchers identifying local cultural practices which may be harnessed to support delivery of breathlessness interventions (e.g. breathing exercises)^64^. Applied health researchers have an important role to play in intervention development and evaluation. However, involvement of social scientists (e.g. ethnographers, psychologists) may be a helpful way to identify other cultural practices for which there may be unexplored health benefits and of involving communities in the development of solutions to the problems that they face.

Implementation of breathlessness interventions becomes more likely if evidence is co-designed and interventions address meaningful outcomes for individuals, families and health systems. We identified evidence that breathlessness negatively impacts on ability to undertake employment. In India and other LMICs where most people lack a social safety net, not working means not earning. Very high prevalence of breathlessness in India indicates that breathlessness may profoundly impact economic wellbeing of individuals and families. Prospective evaluation is needed to explore this issue alongside evaluation of whether breathlessness interventions can mitigate any negative effects.

### Strengths and limitations

Our review has significant strengths. We involved international and national stakeholders with varied experiences of breathlessness and breathlessness management to contribute to evidence development and interpretation. This means that a wide range of views and experiences are included within our review. We also included key evidence developed in HICs and in India and other countries in Asia, to develop evidence relevant to our research questions. The lead researcher (JC) lives in a HIC, therefore offering an ‘outsider’ view of contexts for breathlessness management in India. Interaction of outsider and insider views allowed a dynamic and nuanced interpretation of evidence. Our findings and methods have implications for other LMICs, although we encourage other teams to use our approach to develop evidence for breathlessness research in other country contexts.

Our review also has limitations. Although we included a diverse range of stakeholders, India is a country of 1.5 billion people so some views may be underrepresented. We aimed to include stakeholders from different professional and non-professional backgrounds (e.g. clinical specialty, type of experience, geographic location). However, stakeholders were identified using the networks of co-investigators and some views may not be represented. Additionally, a realist review requires an interpretive approach, therefore researchers with different lived experiences may have reached different conclusions. Nevertheless, we used systematic approaches, transparent methods without conflicting interests, to develop theory for how breathlessness should be understood, identified and address for the context of India.

## Conclusions

Breathlessness is highly prevalent in communities In India, but is commonly undiagnosed and unaddressed. Breathlessness management in India must be part of an integrated approach to healthcare which promotes reversal of avoidable causes and treatment of modifiable causes alongside symptom management. Low-cost, non-pharmacological self-management interventions can be implemented in India, where trusted health workers address health beliefs at the point of delivery.

## Supplementary information

Supplementary File 1

Supplementary File 2

## Data Availability

No datasets were generated or analysed during the current study.
