# Supplementary material for: BREATHLEssness in INDIA (BREATHE-INDIA): realist review to develop explanatory programme theory about breathlessness self-management in India
Source: NPJ Prim Care Respir Med. 2025 Mar 13;35:13. doi: 10.1038/s41533-025-00420-2 (PMC11906595; doi:10.1038/s41533-025-00420-2)
Supplement: Supplementary file 1 [file 41533_2025_420_MOESM1_ESM.docx]

**Supplementary File 1. Search strategy**

Ovid MEDLINE(R) ALL <1946 to July 19, 2023>

 1              Dyspnea, Paroxysmal/ or Dyspnea/

2              breathless*.ti,ab,kw.

3              dyspn*.ti,ab,kw.

4              (short* adj3 breath*).ti,ab,kw.

5              or/1-4 [breathlessness concept]

6              ((non-drug or non-pharmacological or non-medic*) adj3 (intervention* or device* or approach* or treatment* or therapy or therapies)).ti,ab,kw.

7              exp Self-Help Devices/

8              (walker* or rollator* or mobility or self-help or fan*).ti,ab,kw.

9              ((disabled or handicapped or Blue) adj (badge* or park*)).ti,ab,kw.

10           *Disabled Persons/ and *Parking Facilities/

11           (self adj2 (manage* or help)).ti,ab,kw.

12           Cognitive Behavioral Therapy/

13           ((behavior* or behaviour* or cognitive or emotion*) adj2 (therap* or intervention* or approach*)).ti,ab,kw.

14           exp Breathing Exercises/

15           (inspiratory muscle training or RESISTANCE or RESPIRATORY MUSCLE TRAINING).ti,ab,kw.              850579

16           (breathlessness adj2 service*).ti,ab,kw.

17           complex intervention*.ti,ab,kw.

18           (multi adj component intervention*).ti,ab,kw.

19           exp Complementary Therapies/ 242073

20           (meditation or accupressure or mindful* or music therapy or yoga or acupuncture or reiki or "compressed air" or fan or "room air" or "room environment" or "water spray" or (handheld adj fan*)).ti,ab,kw.

21           (multidisciplinary or psychosocial or psychotherapy or biofeedback or "adaption strategies" or "energy conservation" or "activity pacing" or "teaching coping" or relaxation therapy or distraction therapy or exercise or "breathing techniques" or "breathing exercise*" or tai chi).ti,ab,kw.

22           ((walking or mobility or ambulatory) adj2 aid*).ti,ab,kw.

23           ((nondrug or nonpharmacological or nonmedic*) adj3 (intervention* or device* or approach* or treatment* or therapy or therapies)).ti,ab,kw.

24           exp Psychotherapy/

25           Case Management/

26           Integrative Medicine/

27           multi-component.ti,ab,kw.

28           multi-dimension*.ti,ab,kw.

29           pulmonary rehabilitation.mp.

30           self care/ or self-management/

31           or/6-30 [ non medical interventions]

32           5 and 31 [dyspnea AND self management interventions]

33           Developing Countries.sh,kf.

34           (Filter to countries in Asia).

35           ((developing or less* developed or under developed or underdeveloped or middle income or low* income or underserved or under served or deprived or poor*) adj (countr* or nation? or population? or world)).ti,ab.                127851

36           ((developing or less* developed or under developed or underdeveloped or middle income or low* income) adj (economy or economies)).ti,ab.

37           (low* adj (gdp or gnp or gross domestic or gross national)).ti,ab.

38           (low adj3 middle adj3 countr*).ti,ab.

39           (lmic or lmics or third world or lami countr*).ti,ab.

40           transitional countr*.ti,ab.

41           ((high burden or high-burden or countdown) adj countr*).ti,ab.

42           or/33-42 [LMIC concept Sutton 2022]

43           32 and 43

44           (exp Child/ or Adolescent/ or exp Infant/) not exp Adult/ [limit to child only studies]         2098060

46           44 not 45 [removes child only studies]
