# Supplementary material for: BREATHLEssness in INDIA (BREATHE-INDIA): realist review to develop explanatory programme theory about breathlessness self-management in India
Source: NPJ Prim Care Respir Med. 2025 Mar 13;35:13. doi: 10.1038/s41533-025-00420-2 (PMC11906595; doi:10.1038/s41533-025-00420-2)
Supplement: Supplementary file 2 [file 41533_2025_420_MOESM2_ESM.docx]

**Supplementary File 2. Included sources table**

| **Source** | **Contribution to synthesis** | **Author** | **Year** | **Setting** | **Type** | **Design** | **Population** | **Intervention** | **Delivery** | **Domain** |
| --- | --- | --- | --- | --- | --- | --- | --- | --- | --- | --- |
| Expert recommended | High | Brakema et al^[[1]](#endnote-1)^ | 2022 | 'Low resource rural settings' in Uganda, Kyrgyzstan, Vietnam, and Greece | Peer-reviewed journal article | Observational mixed method study | Community members’ households and health-care professionals | NA | NA | Context, beliefs and behaviours |
| Expert recommended | High | Kane et al^^[[2]](#endnote-2)^^ | 2022 | Twenty villages in two states in India | Peer-reviewed journal article | Qualitative | People with breathlessness and family members | NA | NA | Context, beliefs and behaviours |
| Expert recommended | High | Malpass et al^^[[3]](#endnote-3)^^ | 2018 | Primary and secondary care (United Kingdom) | Peer-reviewed journal article | Qualitative | Patients with asthma or chronic obstructive pulmonary disease | Mindfulness-based cognitive therapy (MBCT) | Researcher | Intervention development |
| Expert recommended | High | Spathis et al^^[[4]](#endnote-4)^^ | 2017 | NA | Peer-reviewed journal article | NA | Refractory breathlessness | The Breathing Thinking Functioning clinical model | NA | Intervention development |
| Expert recommended | High | Johnson et al^[[5]](#endnote-5)^ | 2017 | International | Peer-reviewed journal article | Delphi Method | Expert group consultations | NA | NA | Intervention development |
| Expert recommended | High | Habib GM^[[6]](#endnote-6)^ | 2024 | Bangladesh | Peer-reviewed journal article | Qualitative study | People with COPD | Pulmonary Rehabilitation | NA | Intervention delivery |
| Expert recommended | Medium | Johnson et al^^[[7]](#endnote-7)^^ | 2015 | Respiratory, oncology or palliative care clinics at eight UK centres | Peer-reviewed journal article | RCT | Breathlessness in people with malignant lung disease | Three or one breathing technique training sessions | Type of professional (e.g. nurse, physiotherapist, occupational therapist) not stipulated | Intervention development |
| Expert recommended | Medium | Lovell et al, 2019^^[[8]](#endnote-8)^^ | 2019 | Any setting (hospital, community, or outpatient), multiple countries | Peer-reviewed journal article | Systematic review of qualitative or mixed-method studies | Adults experiencing breathlessness and living with advanced illness (including but not limited to COPD, ILD, chronic heart failure, and cancer) | NA | NA | Context, beliefs and behaviours |
| Expert recommended | Medium | McDonnell et al^^[[9]](#endnote-9)^^ | 2022 | Online delivery to partners in India (Pune and Delhi), Sri Lanka, Kyrgyzstan and Uganda | Peer-reviewed journal article | Adapted framework for evaluation of learning and impact | Nurses, physiotherapists, doctors and early career health professionals / researchers. | Combined educational and PR service development concepts with core clinical content | Researchers | Intervention delivery |
| Expert recommended | Medium | Barnes-Harris et al^^[[10]](#endnote-10)^^ | 2021 | Single tertiary hospital in South India | Peer-reviewed journal article | Qualitative | Physicians from oncology, palliative care, cardiology, and respiratory specialties | NA | NA | Context, beliefs and behaviours |
| Expert recommended | Medium | Clark et al^^[[11]](#endnote-11)^^ | 2022 | South Australia | Peer-reviewed journal article | Cross-sectional survey | General population | NA | NA | Context, beliefs and behaviours |
| Iterative | Medium | Pallium India**^^[[12]](#endnote-12)^^** | 2022 | India | Nongovernmental organization website | News item | General population | NA | NA | Context, beliefs and behaviours |
| Iterative | Medium | Yadav et al**^^[[13]](#endnote-13)^^** | 2022 | 28 villages from two rural blocks in the Gorakhpur district of eastern Uttar Pradesh, North India. | Peer-reviewed journal article | Structured questionnaire | Gorakhpur Health and Demographic Surveillance System (GHDSS) cohort of North India. | NA | NA | Context, beliefs and behaviours |
| Iterative | Medium | Advocate Health^^[[14]](#endnote-14)^^ | 2022 | United States | Policy guideline | Policy guideline | Health care providers interacting with  Patients of the Hindu religion and their families | NA | NA | Context, beliefs and behaviours |
| Iterative | Medium | Worthington et al^[[15]](#endnote-15)^ | 2011 | Primary Care, India | Peer-reviewed journal article | Case-based analysis | A woman with somatic symptoms seemingly triggered by psychological stresses associated with social norms and familial cultural expectations. | NA | NA | Context, beliefs and behaviours |
| Iterative | Medium | Gergianaki^[[16]](#endnote-16)^ | 2019 | Low resource settings | Peer-reviewed journal article | Literature review | COPD | Spirituality | NA | Intervention development |
| Iterative | Medium | Silva H^[[17]](#endnote-17)^ | 2020 | India | Peer-reviewed journal article | Literature review | Vascular | Menthol | NA | Intervention development |
| Iterative | Medium | IPCRG^[[18]](#endnote-18)^ | 2023 | Primary care, International | Charitable website | Media | NA | Educational materials | NA | Intervention development |
| Iterative | Medium | Dharmsala^[[19]](#endnote-19)^ | 2023 | India | Media | Review | NA | Ayurveda | NA | Context, beliefs and behaviours |
| Iterative | Medium | News18^^[[20]](#endnote-20)^^ | 2022 | India | Media | Media | NA | Health Literacy | NA | Context, beliefs and behaviours |
| Systematic review | Medium | Lheng et al^[[21]](#endnote-21)^ | 2017 | Low resource settings (Scotland, India and Nepal) | Peer-reviewed journal article | Literature review | Respiratory problems | Hand held fans, breathing techniques, graded exercise and use of low-dose morphine | N/A | Context, beliefs and behaviours |
| Systematic review | Medium | Agnihotri S et al^[[22]](#endnote-22)^ | 2017 | Northern India | Peer-reviewed journal article | RCT | Asthma | Participants in “the Yoga group” received yogic intervention (Asanas, Pranayama and meditation) for 30 min per day, 5 days in a week for 6 months | Not reported | Intervention development |
| Systematic review | Medium | Jeong et al^[[23]](#endnote-23)^ | 2015 | Home | Peer-reviewed journal article | Prospective evaluation | Lung cancer | Caregiver education | Not reported | Intervention development |
| Systematic review | Medium | Gothi D et al^[[24]](#endnote-24)^ | 2011 | India | Peer-reviewed journal article | Literature review | COPD | Pulmonary rehabilitation | NA | Intervention development |
| Systematic review | Medium | Singh et al^[[25]](#endnote-25)^ | 2009 | Hospital outpatient clinic, India | Peer-reviewed journal article | RCT | COPD | Music group subjects listened to their selected music through head phones via a portable audio disc player for 30 minutes in two sessions (morning and afternoon) on the same day. | Not reported | Intervention development |
|  |  |  |  |  |  |  |  |  |  |  |
| Expert recommended | Low | Look et al^[[26]](#endnote-26)^ | 2021 | University of Malaya Medical Centre, Malaysia | Peer-reviewed journal article | RCT | Adult palliative care patients aged 18 years and above and (2) at least one symptom scoring ≥5/10 based on the Edmonton Symptom Assessment Scale (ESAS). | 20 min mindful breathing | Researcher | Intervention development |
| Expert recommended | Low | Pal et al^^[[27]](#endnote-27)^^ | 2022 | Social Media, India | Peer-reviewed journal article | RCT | Apparently healthy adults in context of COVID-19 | Online mindfulness program | Online | Intervention development |
| Expert recommended | Low | Tan et al^^[[28]](#endnote-28)^^ | 2019 | University Malaya Medical Centre in Malaysia | Peer-reviewed journal article | RCT | People with lung cancer, COPD | 20-minute mindful breathing | Respiratory team | Intervention development |
| Expert recommended | Low | Yoeli and McNaughton al^^[[29]](#endnote-29)^^ | 2021 | Literature Review, international | Peer-reviewed journal article | Phenomenological meta-ethnography | COPD | Singing group | NA | Intervention development |
| Expert recommended | Low | Clark et al^^[[30]](#endnote-30)^^ | 2022 | Low resource countries | Peer-reviewed journal article | Literature review | Respiratory problems | NA | NA | Context, beliefs and behaviours |
| Expert recommended | Low | Swan et al^[[31]](#endnote-31)^ | 2019 | Outpatient department, United Kingdom | Peer-reviewed journal article | Feasibility RCT | Respiratory conditions | Handheld fan and/or Calming Hand |  | Intervention development |
| Iterative | Low | Babu et al^^[[32]](#endnote-32)^^ | 2019 | Kerala, India | Peer-reviewed journal article | Cross sectional study | Adult women in the general population | NA | NA | Context beliefs and behaviours |
| Iterative | Low | Raguram^^[[33]](#endnote-33)^^ | 1996 | South India | Peer-reviewed journal article | Case report | 23 year-old female who presents with episodes of anxiety, accompanied by feelings of impending doom, shortness of breath, palpitations, and loss of sensation in her limbs lasting for 15-20 minutes | NA | NA | Context beliefs and behaviours |
| Iterative | Low | VectisKArma^[[34]](#endnote-34)^ | 2023 | India | Media | Media article | NA | Incense burning | NA | Context beliefs and behaviours |
| Iterative | Low | Nagpure AS^[[35]](#endnote-35)^ | 2020 | India | Media | Media article | NA | Household air pollution | NA | Context beliefs and behaviours |
| Iterative | Low | Elf et al^^[[36]](#endnote-36)^^ | 2018 | Low-income urban homes in India | Peer-reviewed journal article | Cross-sectional survey | NA | Indoor air pollution risk factors | NA | Context beliefs and behaviours |
| Iterative | Low | The Eco Muslim^[[37]](#endnote-37)^ | 2023 | NA | Media | Media article | NA | Incense burning | NA | Context beliefs and behaviours |
| Systematic review | Low | Lee et al^[[38]](#endnote-38)^ | 2022 | Home and Hospital, South Korea | Peer-reviewed journal article | RCT | COPD | lung-conduction exercises (LCE) 5 times a week plus medication | Doctor | Intervention development |
| Systematic review | Low | Zaki et al^[[39]](#endnote-39)^ | 2023 | Outpatient unit, India | Peer-reviewed journal article | RCT | ILD | Pulmonary Rehabilitation & Inspiratory muscle training for 21 minutes each day using device | Physiotherapist | Intervention development |
| Systematic review | Low | Bains et al^[[40]](#endnote-40)^ | 2022 | Outpatient unit, India | Peer-reviewed journal article | Experimental study | COPD | Muscle energy technique and Joint manipulation for 2 times a week for 3 weeks | Physiotherapist | Intervention development |
| Systematic review | Low | Ahmed et al^[[41]](#endnote-41)^ | 2020 | Outpatient unit and home, Bangladesh | Peer-reviewed journal article | Experimental study | COPD | Home based pulmonary rehabilitation: Pursed Lip breathing, Diaphragmatic breathing, Lower Extremity Endurance Training - for 30mins, 2x a day, for 60 days. | Doctor | Intervention development |
| Systematic review | Low | Yu et al^[[42]](#endnote-42)^ | 2022 | Hospital, China | Peer-reviewed journal article | RCT | COPD | Mindfulness Behaviour Intervention Combined with Progressive Breathing Training | A multidisciplinary COPD management team | Intervention development |
| Systematic review | High | Zeb et al^[[43]](#endnote-43)^ | 2021 | Hospital, Pakistan | Peer-reviewed journal article | Phenomenological inquiry | COPD | Experiences of self care and role of the family | NA | Context, beliefs and behaviours |
| Systematic review | Low | Molassiotis et al^[[44]](#endnote-44)^ | 2021 | Hospital and home, Vietnam | Peer-reviewed journal article | RCT | Lung Cancer | Qigong: body posture, breathing practise & meditation performed in synchrony (90 min training, 2x per week for 1st 2 weeks, then for last 4 weeks practise at home for 30mins a day, 5x a week) | Nurse | Intervention development |
| Systematic review | Low | Priya et al^[[45]](#endnote-45)^ | 2021 | Outpatient unit and home, India | Peer-reviewed journal article | Quasi-experimental study | COPD | Home based pulmonary rehabilitation for 30 min (Breathing exercise, Pursed lip breathing, upper & lower limb training, slow walking) under supervision. Follow up at home for 6 weeks | Not reported | Intervention development |
| Systematic review | Low | Lu et al^[[46]](#endnote-46)^ | 2020 | Inpatient unit and home, China | Peer-reviewed journal article | Experimental study | AECOPD | Zheng’s Supine Rehabilitation Exercise (ZSRE) (Pull up, bridging, air stepping) 3x day, 15 – 20 repetitions each time- until 8 weeks of discharge. Submit video twice a week. | Not reported | Intervention development |
| Systematic review | Low | Lee et al^[[47]](#endnote-47)^ | 2020 | Hospital and home, South Korea | Peer-reviewed journal article | Experimental study | COPD | Home based Pulmonary rehabilitation– exercise, education & physical therapy for 1 hr. Unsupervised HBPR- 30min a day, 3x week for 8 weeks. Weekly calls – encourage & examine. Diaries – review compliant and non-compliant. | Doctor | Intervention development |
| Systematic review | Low | Ji et al^[[48]](#endnote-48)^ | 2019 | Hospital and home, South Korea | Peer-reviewed journal article | RCT | Non-small cell lung cancer | Mobile Health Management Platform–Based Pulmonary Rehabilitation | Doctor and mobile application | Intervention delivery |
| Systematic review | Low | Khan^[[49]](#endnote-49)^ | 2019 | Public health facilities, Pakistan | Peer-reviewed journal article | cRCT | COPD | Integrated COPD care | Doctors and allied staff | Intervention delivery |
| Systematic review | Low | Sutanto et al^[[50]](#endnote-50)^ | 2019 | Hospital, Indonesia | Peer-reviewed journal article | RCT | COPD | Hospital-based outpatient exercise training program and a Wii Fit videogame program | Doctor and nurse | Intervention development |
| Systematic review | Low | Tarigan et al^[[51]](#endnote-51)^ | 2019 | Hospital outpatient, Indonesia | Peer-reviewed journal article | Quasi-experimental study | COPD | Upper Limb Training with Breathing Maneuver | Physiotherapist and physician | Intervention development |
| Systematic review | Low | Cui et al^[[52]](#endnote-52)^ | 2019 | Hospital, China | Peer-reviewed journal article | RCT | COPD | Multidisciplinary respiratory rehabilitation | Multidisciplinary team | Intervention development |
| Systematic review | Low | Tarigan et al^[[53]](#endnote-53)^ | 2018 | Hospital outpatient, Indonesia | Peer-reviewed journal article | Quasi-experimental study | COPD | Lower-Limb Endurance Training | Physiotherapist | Intervention development |
| Systematic review | Low | Widyasttuti et al^[[54]](#endnote-54)^ | 2018 | Hospital outpatient, Indonesia and home | Peer-reviewed journal article | RCT | COPD | Home pedometer assisted physical activity | Doctor | Intervention development |
| Systematic review | Low | Puspawati NL^[[55]](#endnote-55)^ | 2017 | Hospital, Indonesia | Peer-reviewed journal article | RCT | Lung cancer | Airflow stimulation from hand-held fan A wet damp cloth was used to wipe participant's face without drying, and then airflow from hand-held fan was given. | Not reported | Intervention development |
| Systematic review | Low | Ranjita et al^[[56]](#endnote-56)^ | 2016 | Outpatient clinic, India | Peer-reviewed journal article | RCT | COPD | Yoga (Yogic counselling for stress management, chanting, asanas, pranayama, relaxation technics, meditation and lectures on yogic lifestyle and philosophy. | Yoga therapist | Intervention development |
| Systematic review | Low | Kang et al^[[57]](#endnote-57)^ | 2016 | Outpatient clinic, South Korea | Peer-reviewed journal article | Experimental study | COPD | Diaphragmatic respiration exercise therapists place hands on patient’s abdomen and induce patients to inhale slowly and deeply through their nose, applying resistance to the abdomen. | Physiotherapist | Intervention development |
| Systematic review | Low | Deepak et al^[[58]](#endnote-58)^ | 2014 | Outpatient clinic, India | Peer-reviewed journal article | Experimental study | AECOPD | Pulmonary rehabilitation consists of patient assessment, exercise testing, exercise training, education, nutrition and psycho-social rehabilitation. | Pulmonologist | Intervention development |
| Systematic review | Low | Ali et al^[[59]](#endnote-59)^ | 2014 | Inpatient hospital and home, India | Peer-reviewed journal article | Experimental study | AECOPD | Pulmonary rehabilitation; Self-management education, and exercises - walking, biking and resistance exercises | Not clear | Intervention development |
| Systematic review | Low | Gupta et al^[[60]](#endnote-60)^ | 2014 | Tertiary care institution, India | Peer-reviewed journal article | Experimental study | COPD | Pranayam for 30 minutes, twice per day | Yoga instructors | Intervention development |
| Systematic review | Low | Jingar et al^[[61]](#endnote-61)^ | 2013 | India | Peer-reviewed journal article | Cross-sectional survey | COPD | NA | Physiotherapists | Context, beliefs and behaviours |
| Systematic review | Low | Kyung et al^[[62]](#endnote-62)^ | 2008 | Outpatient clinic, South Korea | Peer-reviewed journal article | Experimental study | COPD | Pulmonary rehabilitation - breathing exercise, upper-limb exercises and IMT | Nurse | Intervention development |
| Systematic review | Low | Oh EGl^[[63]](#endnote-63)^ | 2013 | Hospital, Home, South Korea | Peer-reviewed journal article | Experimental study | COPD | The 8-week home-based pulmonary rehabilitation program consisted of four components: (a) education, (b) inspiratory muscle training, (c) exercise training, and (d) psychosocial components that include relaxation and telephone calls. | Researcher | Intervention development |
| Systematic review | Low | Singh et al^[[64]](#endnote-64)^ | 2003 | Hospital and home, India | Peer-reviewed journal article | RCT | COPD | Rehabilitation included walking, breathing exercises, postural drainage, controlled coughing and changes in life style activities. | Not reported | Intervention development |
| Systematic review | Low | Azeem et al^[[65]](#endnote-65)^ | 2018 | Hospital and home, Pakistan | Peer-reviewed journal article | RCT | COPD | Breathing exercises including compressed lip respiration and diaphragmatic work | Nurse | Intervention development |
| Systematic Review | Low | Liu et al^[[66]](#endnote-66)^ | 2019 | Hospital and home, China | Peer-reviewed journal article | RCT | COPD | Group singing therapy (relaxation, respiratory exercises, vocalization exercises, singing exercises) | Music therapist, community nurse. | Intervention development |
| Systematic review | Low | Lin et al^[[67]](#endnote-67)^ | 2019 | Hospital and home, China | Peer-reviewed journal article | RCT | COPD | Breathing-based walking | Researcher | Intervention development |
| Systematic review | Low | Wei-Li Gu et al^[[68]](#endnote-68)^ | 2018 | Hospital and Home, China | Peer-reviewed journal article | RCT | COPD | Breathing training method based on expiratory airflow limitation and impaired inspiratory muscle function in COPD patients with rapid deep inspiration and prolonged expiration | Researcher | Intervention development |
| Systematic review | Low | Li et al^[[69]](#endnote-69)^ | 2018 | Hospital and home, China | Peer-reviewed journal article | RCT | COPD | Long-term home-based Liuzijue exercise combined with clinical guidance | Integrative Medicine Specialist | Intervention development |
| Systematic review | Low | Thokchom^[[70]](#endnote-70)^ | 2018 | Hospital and home, India | Peer-reviewed journal article | Literature review | COPD | Yoga | Not specified | Intervention development |
| Systematic review | Low | Ranjita et al^[[71]](#endnote-71)^ | 2016 | Workplace and home, India | Peer-reviewed journal article | RCT | COPD | Integrated yoga practices specially designed for COPD and all intellectual abilities | Yoga instructors | Intervention development |
| Systematic review | Low | Wu et al^[[72]](#endnote-72)^ | 2018 | Home and hospital, China | Peer-reviewed journal article | RCT | COPD | Liuzijue | Liuzijuei Instructor | Intervention development |
| Systematic review | Low | Liu et al^[[73]](#endnote-73)^ | 2015 | Hospital, China | Peer-reviewed journal article | RCT | COPD (men with) | Deep-breathing exercise with oxygen inhalation | Researcher | Intervention development |
| Systematic review | Low | Xiao et al^[[74]](#endnote-74)^ | 2015 | Hospital and home, China | Peer-reviewed journal article | RCT | COPD | Liuzijue qigong | Trained therapist and audio-visual material | Intervention development |
| Systematic review | Low | Leelarungrayub et al^[[75]](#endnote-75)^ | 2018 | Public health centre, Thailand | Peer-reviewed journal article | Experimental study | COPD | Lung volume therapy, Slow-deep-breathing-technique, respiratory muscle training | Physical therapist | Intervention development |
| Systematic review | Low | Sakhaei et al^[[76]](#endnote-76)^ | 2018 | Hospital, Iran | Peer-reviewed journal article | RCT | COPD | Posterolateral fusion with pedicle fixation, cognitive intervention and exercises | Nurse | Intervention development |
| Systematic review | Low | Kzar et al^[[77]](#endnote-77)^ | 2017 | Hospital, Iraq | Peer-reviewed journal article | Experimental study | COPD | Inspiratory muscle strength training | Researcher | Intervention development |
| Systematic review | Low | Leelarungrayub et al^[[78]](#endnote-78)^ | 2017 | Public health centre, Thailand | Peer-reviewed journal article | RCT | COPD | Respiratory muscle training | Physical therapist | Intervention development |
| Systematic review | Low | Heydari et al^[[79]](#endnote-79)^ | 2015 | Hospital, Iran | Peer-reviewed journal article | RCT | COPD | Inspiratory Resistive Muscle Training | U/K | Intervention development |
| Systematic review | Low | Fukuoka et al^[[80]](#endnote-80)^ | 2016 | Hospital, Japan | Peer-reviewed journal article | RCT | COPD | Laughing yoga | Yoga therapist | Intervention development |
| Iterative | Low | Bhandari et al^[[81]](#endnote-81)^ | 2007 | Rural places, India | Policy document | Government Report | Rural health Infrastructure | Community healthcare workers | NA | Implementation |
| Iterative | Medium | Mishra et al^[[82]](#endnote-82)^ | 2010 | Community, India | Peer-reviewed journal article | Qualitative study | ASHA workers | Delivery of healthcare | NA | Implementation |
| Iterative | Low | Srivastava et al^[[83]](#endnote-83)^ | 2022 | Community, India | Peer-reviewed journal article | Case study | Students | Effective communication in pandemic using ASHAs as example | NA | Intervention development |
| Iterative | Low | Karol et al^[[84]](#endnote-84)^ | 2014 | Rural areas, Rajasthan, India | Peer-reviewed journal article | Evaluative study | ASHA workers | Knowledge on reproductive and child health | NA | Intervention development |
| **Iterative** | Low | Bajpai et al^[[85]](#endnote-85)^ | 2011 | Community, India | Book | Survey | ASHA workers | Questionnaires given to ASHA workers | NA | Knowledge and attitude |
| Iterative | Low | Paul et al^[[86]](#endnote-86)^ | 2020 | India | Peer-reviewed journal article | Survey | ASHA workers | Questionnaires circulated to women across states and union territories of India | NA | Knowledge and attitude |
| Iterative | Low | Holt et al^[[87]](#endnote-87)^ | 2021 | Community, Gujarat, India | Peer-reviewed journal article | Qualitative study | Women perspective on Contraception | Eight focus group discussion with rural women | Rural women | Knowledge and attitude |
| Iterative | Low | Singh et al^[[88]](#endnote-88)^ | 2017 | Community, India | Peer-reviewed journal article | Mixed method study | DOT providers - ASHAs | Quantitative component assessed the knowledge and practices, and focus-group discussions explored the attitude and barriers | NA | Knowledge and attitude |
| Iterative | Low | Jain et al^[[89]](#endnote-89)^ | 2020 | Community, India | Peer-reviewed journal article | Qualitative study | Healthcare workers | Focus group discussion on Childhood vaccination | NA | Knowledge and attitude |
| Iterative | Low | Bhatt et al^[[90]](#endnote-90)^ | 2014 | Rural Community, India | Peer-reviewed journal article | Literature review | Diabetes care | Review on Diabetes care | ASHA workers | Knowledge and attitude |
| Iterative | Medium | Scott et al^[[91]](#endnote-91)^ | 2010 | Community, India | Peer-reviewed journal article | Case study (Prospective) | ASHA worker – Success rate | Interviews and focus groups with ASHAs, HP and community members as well as non-participant observation at public health centres | NA | Intervention development |
| Iterative | Low | Ramani et al^[[92]](#endnote-92)^ | 2013 | Community, India | Peer-reviewed journal article | Qualitative study | Students & In-service professionals | In depth interviews – Rural services (Attitudes and Incentives) | NA | Knowledge and attitude |
| Iterative | Medium | Lall et al^[[93]](#endnote-93)^ | 2019 | Community, India | Peer-reviewed journal article | Mixed method | Health professional | In depth interview – on delivery of care in rural areas | NA | Intervention development |
| Iterative | Low | Kanungo et al^[[94]](#endnote-94)^ | 2015 | Community, India | Peer-reviewed journal article | Cross sectional study | Healthcare seeking in community | Interviews | NA | Knowledge and attitude |
| Iterative | Low | Starfield et al^[[95]](#endnote-95)^ | 2005 | India | Peer-reviewed journal article | Evidence based study | Primary healthcare workers | Evidence – Prevention of illness and death | NA | Intervention Development |
| Iterative | Low | Srinidhi et al^[[96]](#endnote-96)^ | 2021 | India | Peer-reviewed journal article | Literature review | ASHA workers | Review – Implementing M-CAT tool training, hands-on assistance and regular meetings | ASHA workers | Knowledge and attitude |
| Iterative | Medium | Bansal et al^[[97]](#endnote-97)^ | 2014 | India | Peer-reviewed journal article | Literature review | Education system | Review - Blended learning | NA | Intervention development |
| Iterative | Low | Joshi et al^[[98]](#endnote-98)^ | 2014 | LMIC | Peer-reviewed journal article | Systematic review | Management of Non-Communicable disease | Review | NA | Knowledge and attitude |
| Iterative | Low | Shah et al^[[99]](#endnote-99)^ | 2019 | Community, India | Peer-reviewed journal article | Cross sectional study | ASHA & Anganwadi workers | Knowledge and attitudes for mental illness | NA | Intervention development |
| Iterative | Low | Srivastava et al^[[100]](#endnote-100)^ | 2015 | India | Peer-reviewed journal article | Literature review | Skill development | Vocational training and education | Schools and colleges | Intervention development |
| Iterative | Low | Kidd et al^[[101]](#endnote-101)^ | 2021 |  | Book | Review | Improving Health Systems | Health needs and challenges | NA | Knowledge |
| Iterative | Low | Hunter et al^[[102]](#endnote-102)^ | 2014 | India | Peer-reviewed journal article | Systematic review | Promotion of maternal health | Finance and promoting Maternal health | NA | Intervention development |
| Iterative | Low | Husain et al^[[103]](#endnote-103)^ | 2014 | India | Peer-reviewed journal article | Literature review | Rural Health Mission | Rural health infrastructure and facilities | NA | Knowledge and attitude |
| Iterative | Low | Kaysin et al^[[104]](#endnote-104)^ | 2017 | India | Peer-reviewed journal article | Cross sectional study | ASHA workers | Maternal health services | NA | Knowledge an |

1. Brakema E et al. Mapping low-resource context to prepare for lung health interventions in four countries (FRESH AIR): a mixed-methods study. *Lancet Global Health* 2022;10(1), E63-76 [↑](#endnote-ref-1)
2. Kane S, Joshi M, Desai S, Mahal A, McPake B. People's care seeking journey for a chronic illness in rural India: Implications for policy and practice. Soc Sci Med. 2022 Nov;312:115390. doi: 10.1016/j.socscimed.2022.115390. Epub 2022 Sep 23. PMID: 36167024; PMCID: PMC9582193. [↑](#endnote-ref-2)
3. Malpass A et al. Understanding changes in dyspnoea perception in obstructive lung disease after mindfulness training. *BMJ Open Respiratory Research* 2018;5:e000309. [↑](#endnote-ref-3)
4. Spathis A et al. The Breathing, Thinking, Functioning clinical model: a proposal to facilitate evidence-based breathlessness management in chronic respiratory disease. *Primary Care Respiratory Medicine* 2017 DOI: 10.1038/s41533-017-0024-z. [↑](#endnote-ref-4)
5. Johnson M, Yorke J, Hansen-Flaschen J et al. Towards an expert consensus to delineate a clinical syndrome of chronic breathlessness. *European Respiratory Journal,* 2017;49. DOI: 10.1183/13993003.02277-2016. [↑](#endnote-ref-5)
6. Habib GMM, Uzzaman N, Rabinovich R et al. Exploring the perceptions of patients with chronic respiratory diseases and their insights into pulmonary rehabilitation in Bangladesh. *J of Global Health,* 2024. DOI: 10.7189/jogh.14.04036. [↑](#endnote-ref-6)
7. Johnson M et al. A randomised controlled trial of three or one breathing technique training sessions for breathlessness in people with malignant lung disease. *BMC Medicine* 2015;13:213. [↑](#endnote-ref-7)
8. Lovell N et al. Control and context are central for people with advanced illness experiencing breathlessness: a systematic review and thematic synthesis. *Journal of Pain and Symptom Management* 2019;57(1):140-155 [↑](#endnote-ref-8)
9. McDonell J et al. The RECHARGE-IPCRG ‘Teach the Teacher’ programme: building capacity for pulmonary rehabilitation in low- and middle-income countries. *Journal of Global Health Reports* Nov 2022 [↑](#endnote-ref-9)
10. Barnes-Harris MMM, Daniel S, Venkateswarn C et al. How Physicians in South India Recognize, Assess, and Manage People with Chronic Breathlessness Syndrome: A Thematic Analysis. *Indian J Palliat Care,* 2021;27(1):54-61. [↑](#endnote-ref-10)
11. Clark, J., Chang, S., Kinchin, I. *et al.* Lower workforce participation is associated with more severe persisting breathlessness. *BMC Pulm Med* **22**, 93 (2022). https://doi.org/10.1186/s12890-022-01861-y [↑](#endnote-ref-11)
12. Yadav, R.; Zaman, K.; Mishra, A.; Reddy, M.M.; Shankar, P.; Yadav, P.; Kumar, K.; Kant, R. Health Seeking Behaviour and Healthcare Utilization in a Rural Cohort of North India. Healthcare 2022, 10, 757. https://doi.org/10.3390/healthcare10050757 [↑](#endnote-ref-12)
13. Yadav, R.; Zaman, K.; Mishra, A.; Reddy, M.M.; Shankar, P.; Yadav, P.; Kumar, K.; Kant, R. Health Seeking Behaviour and Healthcare Utilization in a Rural Cohort of North India. Healthcare 2022, 10, 757. https://doi.org/10.3390/healthcare10050757 [↑](#endnote-ref-13)
14. Advocate Health. Guidelines for healthcare providers interacting with patients of the Hindu religion and their families. 2022. Available from: <https://www.advocatehealth.com/assets/documents/faith/cghindu.pdf>. Accessed 12.12.222. [↑](#endnote-ref-14)
15. Worthington RP, Gogne A. Cultural aspects of primary healthcare in india: A case- based analysis. Asia Pac Fam Med. 2011 Jun 16;10(1):8. doi: 10.1186/1447-056X-10-8. PMID: 21679415; PMCID: PMC3130647. [↑](#endnote-ref-15)
16. Gergianaki I, Kampouraki M, Williams S, Tsiligianni I. Assessing spirituality: is there a beneficial role in the management of COPD? NPJ Prim Care Respir Med. 2019 May 28;29(1):23. doi: 10.1038/s41533-019-0134-x. PMID: 31138810; PMCID: PMC6538715. [↑](#endnote-ref-16)
17. Silva H. Current Knowledge on the Vascular Effects of Menthol. Front Physiol. 2020 Apr 7;11:298. doi: 10.3389/fphys.2020.00298. Erratum in: Front Physiol. 2020 Oct 20;11:602231. PMID: 32317987; PMCID: PMC7154148. [↑](#endnote-ref-17)
18. International Primary Care Respiratory Group. How We Breathe. 2023. Available from: <https://www.ipcrg.org/howwebreathe> [↑](#endnote-ref-18)
19. Dharmsala. The concept of balance in Ayurveda. 2023. Available from: <https://www.dharmsala.com/blogs/tea-guide/the-concept-of-balance-in-ayurveda-a-definitive-guide-for-the-lay-person> [↑](#endnote-ref-19)
20. News18. COVID Has Exposed the Low Health Literacy in India. We Must Up the Communication Game. Available from: <https://www.news18.com/news/opinion/covid-has-exposed-the-low-health-literacy-in-india-we-must-up-the-communication-game-4693484.html>. Accessed 25.07.2023. [↑](#endnote-ref-20)
21. Leng ME, Daniel S, Munday D. Respiratory problems in low-resource settings. Current opinion in supportive and palliative care. 2017 Sep 1;11(3):174-8. [↑](#endnote-ref-21)
22. Agnihotri S, Kant S, Mishra SK, Verma A. Assessment of significance of Yoga on quality of life in asthma patients: A randomized controlled study. Ayu. 2017 Jan;38(1-2):28. [↑](#endnote-ref-22)
23. Jeong JH, Yoo WG. Effect of caregiver education on pulmonary rehabilitation, respiratory muscle strength and dyspnea in lung cancer patients. Journal of Physical Therapy Science. 2015;27(6):1653-4. [↑](#endnote-ref-23)
24. Gothi D, Joshi JM. Pulmonary rehabilitation in resource poor settings. Indian Journal of Chest Diseases and Allied Sciences. 2011 Jul 1;53(3):163. [↑](#endnote-ref-24)
25. Singh VP, Rao V, V P, RC S, K KP. Comparison of the effectiveness of music and progressive muscle relaxation for anxiety in COPD—a randomized controlled pilot study. Chronic respiratory disease. 2009 Nov;6(4):209-16. [↑](#endnote-ref-25)
26. Look M et al. Symptom reduction in palliative care from single session mindful breathing: a randomised controlled trial. *BMJ Supportive and Palliative Care.* 2021:11:433-439. [↑](#endnote-ref-26)
27. Pal A et al. Effect of an online mindfulness program on stress in Indian adults during COVID-19 pandemic: a randomised controlled preliminary study. *Indian J Psychiatry* 2022;64(4):401-407. [↑](#endnote-ref-27)
28. Tan S et al. The effect of a 20-minute mindful breathing on the rapid reduction of dyspnoea at rest in patients with lung diseases: a randomised controlled trial. *Journal of Pain and Symptom Management* 2019 doi: 10.1016/j.jpainsymman.2019.01.009. [↑](#endnote-ref-28)
29. Yoeli H et al. ‘To more than I can be’: a phenomenological meta-ethnography of singing groups for people with chronic obstructive pulmonary disease. *Health*, 2021;*25*(5), pp.574-595. [↑](#endnote-ref-29)
30. Clark J, Kochovska S, Currow DC. Burden of respiratory problems in low-income and middle-income countries. Curr Opin Support Palliat Care. 2022 Dec 1;16(4):210-215. doi: 10.1097/SPC.0000000000000615. Epub 2022 Sep 13. PMID: 36102933. [↑](#endnote-ref-30)
31. Swan F, English A, Allgar V et al. The Hand-Held Fan and the Calming Hand for People With Chronic Breathlessness: A Feasibility Trial. *J of Pain and Symptom Manage,* 2019;57(6):1051-1061. [↑](#endnote-ref-31)
32. Babu AR, Sreedevi A, John A, Krishnapillai V. Prevalence and Determinants of Somatization and Anxiety among Adult Women in an Urban Population in Kerala. Indian J Community Med. 2019 [↑](#endnote-ref-32)
33. Raguram R, Weiss MG, Channabasavanna SM, Devins GM. Stigma, depression, and somatization in South India. Am J Psychiatry. 1996 Aug;153(8):1043-9. doi: 10.1176/ajp.153.8.1043. PMID: 8678173. [↑](#endnote-ref-33)
34. Vectis Karma. 14 Reasons to burn Incense and the benefits. 2023. Available from: https://www.vectiskarma.co.uk/Benefits-of-Burning-Incense [↑](#endnote-ref-34)
35. Nagpure AS. WRI India. 2020. Available from: Household Air Pollution from Solid Fuels and its Invisible Solution. Accessed 04.08.2023. [↑](#endnote-ref-35)
36. Elf JL, Kinikar A, Khadse S, Mave V, Suryavanshi N, Gupte N, Kulkarni V, Patekar S, Raichur P, Breysse PN, Gupta A, Golub JE. Sources of household air pollution and their association with fine particulate matter in low-income urban homes in India. J Expo Sci Environ Epidemiol. 2018 Jun;28(4):400-410. doi: 10.1038/s41370-018-0024-2. Epub 2018 May 23. PMID: 29789668; PMCID: PMC6013356. [↑](#endnote-ref-36)
37. The Eco Muslim. Indian Incense Heals Health And Environment (Oud Al-Hind) . 2023. Available from: https://www.theecomuslim.co.uk/2011/10/indian-incense-heals-health-and.html [↑](#endnote-ref-37)
38. Lee SW, Park JJ, Lyu YR, Lee EJ, Kim SY, Kang W, Son JW, Jung IC, Park YC. The effect of lung-conduction exercise in chronic obstructive pulmonary disease: Randomized, assessor-blind, multicenter pilot trial. Medicine. 2022 Jan 1;101(3). [↑](#endnote-ref-38)
39. Zaki S, Moiz JA, Mujaddadi A, Ali MS, Talwar D. Does inspiratory muscle training provide additional benefits during pulmonary rehabilitation in people with interstitial lung disease? A randomized control trial. Physiotherapy Theory and Practice. 2023 Mar 4;39(3):518-28. [↑](#endnote-ref-39)
40. Bains D, Chahal A, Shaphe MA, Kashoo FZ, Ali T, Alghadir AH, Khan M. Effects of Muscle Energy Technique and Joint Manipulation on Pulmonary Functions, Mobility, Disease Exacerbations, and Health-Related Quality of Life in Chronic Obstructive Pulmonary Disease Patients: A Quasiexperimental Study. BioMed Research International. 2022 Jul 30;2022. [↑](#endnote-ref-40)
41. Ahmed NU, Begum S, Ali T, Suhana M. Home Based Pulmonary Rehabilitation on Oxygenation Status, Dyspnoea and Fatigue in Stable Patients with COPD. Mymensingh Medical Journal: MMJ. 2020 Apr 1;29(2):424-30. [↑](#endnote-ref-41)
42. Yu S, Fan H. Analysis of the Effect of Mindfulness Behaviour Intervention Combined with Progressive Breathing Training on Pulmonary Function Rehabilitation in Patients with Chronic Obstructive Pulmonary Disease. Emergency Medicine International. 2022 Aug 27;2022. [↑](#endnote-ref-42)
43. Zeb H, Younas A, Ahmed I, Ali A. Self‐care experiences of Pakistani patients with COPD and the role of family in self‐care: a phenomenological inquiry. Health & Social Care in the Community. 2021 Sep;29(5):e174-83. [↑](#endnote-ref-43)
44. Molassiotis A, Vu DV, Ching SS. The effectiveness of Qigong in managing a cluster of symptoms (Breathlessness-Fatigue-Anxiety) in patients with lung cancer: a randomized controlled trial. Integrative Cancer Therapies. 2021 Apr;20:15347354211008253. [↑](#endnote-ref-44)
45. Priya N, Isaac BT, Thangakunam B, Christopher DJ. Effect of home-based pulmonary rehabilitation on health-related quality of life, lung function, exercise tolerance, and dyspnoea in chronic obstructive pulmonary disorder patients in a tertiary care centre in South India. Lung India: Official Organ of Indian Chest Society. 2021 May;38(3):211. [↑](#endnote-ref-45)
46. Lu H, Liu N, Hu JY, Wang X, Li Y, Song M, Zhong LH, He W, Chen R, Zheng Z. The effectiveness, safety and compliance of Zheng’s supine rehabilitation exercise as a rehabilitation programme among elderly patients with AECOPD. The Clinical Respiratory Journal. 2020 Jun;14(6):533-40. [↑](#endnote-ref-46)
47. Lee JH, Lee HY, Jang Y, Lee JS, Oh YM, Lee SD, Lee SW. Efficacy of unsupervised home-based pulmonary rehabilitation for patients with chronic obstructive pulmonary disease. International Journal of Chronic Obstructive Pulmonary Disease. 2020 Sep 28:2297-305. [↑](#endnote-ref-47)
48. Ji W, Kwon H, Lee S, Kim S, Hong JS, Park YR, Kim HR, Lee JC, Jung EJ, Kim D, Choi CM. Mobile health management platform–based pulmonary rehabilitation for patients with non–small cell lung cancer: Prospective clinical trial. JMIR mHealth and uHealth. 2019 Jun 21;7(6):e12645. [↑](#endnote-ref-48)
49. Khan MA, Khan N, Walley JD, Khan MA, Hicks J, Ahmed M, Sheikh FI, Ali M, Manzoor F, Khan HJ. Effectiveness of delivering integrated COPD care at public healthcare facilities: a cluster randomised trial in Pakistan. BJGP open. 2019 Apr 1;3(1). [↑](#endnote-ref-49)
50. Sutanto YS, Makhabah DN, Aphridasari J, Doewes M, Ambrosino N. Videogame assisted exercise training in patients with chronic obstructive pulmonary disease: A preliminary study. Pulmonology. 2019 Sep 1;25(5):275-82. [↑](#endnote-ref-50)
51. Tarigan AP, Ananda FR, Pandia P, Sinaga BY, Maryaningsih M, Anggriani A. The impact of upper limb training with breathing manoeuvre in lung function, functional capacity, dyspnoea scale, and quality of life in patient with stable chronic obstructive of lung disease. Open Access Macedonian Journal of Medical Sciences. 2019 Feb 2;7(4):567. [↑](#endnote-ref-51)
52. Cui L, Liu H, Sun L. Multidisciplinary respiratory rehabilitation in combination with non-invasive positive pressure ventilation in the treatment of elderly patients with severe chronic obstructive pulmonary disease. Pakistan Journal of Medical Sciences. 2019 Mar;35(2):500. [↑](#endnote-ref-52)
53. Tarigan AP, Pandia P, Mutiara E, Pradana A, Rhinsilva E, Efriyandi E. Impact of lower-limb endurance training on dyspnea and lung functions in patients with COPD. Open Access Macedonian Journal of Medical Sciences. 2018 Dec 12;6(12):2354. [↑](#endnote-ref-53)
54. Widyastuti K, Makhabah DN, Setijadi AR, Sutanto YS, Ambrosino N. Benefits and costs of home pedometer assisted physical activity in patients with COPD. A preliminary randomized controlled trial. Pulmonology. 2018 Jul 1;24(4):211-8. [↑](#endnote-ref-54)
55. Puspawati NL, Sitorus R, Herawati T. Hand-held fan airflow stimulation relieves dyspnea in lung cancer patients. Asia-Pacific journal of oncology nursing. 2017 Apr 1;4(2):162-7. [↑](#endnote-ref-55)
56. Ranjita R, Hankey A, Nagendra HR, Mohanty S. Yoga-based pulmonary rehabilitation for the management of dyspnoea in coal miners with chronic obstructive pulmonary disease: a randomized controlled trial. Journal of Ayurveda and integrative medicine. 2016 Jul 1;7(3):158-66. [↑](#endnote-ref-56)
57. Kang JI, Jeong DK, Choi H. The effects of breathing exercise types on respiratory muscle activity and body function in patients with mild chronic obstructive pulmonary disease. Journal of physical therapy science. 2016;28(2):500-5. [↑](#endnote-ref-57)
58. Deepak TH, Mohapatra PR, Janmeja AK, Sood P, Gupta M. Outcome of pulmonary rehabilitation in patients after acute exacerbation of chronic obstructive pulmonary disease. Indian J Chest Dis Allied Sci. 2014 Jan 1;56(1):7-12. [↑](#endnote-ref-58)
59. Ali MS, Talwar D, Jain SK. The effect of a short-term pulmonary rehabilitation on exercise capacity and quality of life in patients hospitalised with acute exacerbation of chronic obstructive pulmonary disease. Indian J Chest Dis Allied Sci. 2014 Jan 1;56(1):13-9. [↑](#endnote-ref-59)
60. Gupta A, Gupta R, Sood S, Arkham M. Pranayama for treatment of chronic obstructive pulmonary disease: Results from a randomized, controlled trial. Integrative Medicine: A Clinician's Journal. 2014 Feb;13(1):26. [↑](#endnote-ref-60)
61. Jingar A, Alaparthi GK, Vaishali K, Krishnan S, Unnikrishnan B. Clinical management practices adopted by physiotherapists in India for chronic obstructive pulmonary disease: A national survey. Lung India: Official Organ of Indian Chest Society. 2013 Apr;30(2):131. [↑](#endnote-ref-61)
62. Kyung KA, Chin PA. The effect of a pulmonary rehabilitation programme on older patients with chronic pulmonary disease. Journal of clinical nursing. 2008 Jan;17(1):118-25. [↑](#endnote-ref-62)
63. Oh EG. The effects of home-based pulmonary rehabilitation in patients with chronic lung disease. International journal of nursing studies. 2003 Nov 1;40(8):873-9. [↑](#endnote-ref-63)
64. Singh V, Khandelwal DC, Khandelwal R, Abusaria S. Pulmonary rehabilitation in patients with chronic obstructive pulmonary disease. Indian Journal of Chest Diseases and Allied Sciences. 2003 Jan 1;45(1):13-8. [↑](#endnote-ref-64)
65. Azeem M, Harhad M and Chaudry AR. Study of efficacy of breathing exercises in patients with chronic obstructive pulmonary disease in a tertiary care hospital. *Indo J of Pharm Sci,* 219;06(04):8683-8686. [↑](#endnote-ref-65)
66. Liu H, Song M, Zhai ZH, Shi RJ, Zhou XL. Group singing improves depression and life quality in patients with stable COPD: a randomized community-based trial in China. Quality of Life Research. 2019 Mar 15;28:725-35. [↑](#endnote-ref-66)
67. Lin FL, Yeh ML, Lai YH, Lin KC, Yu CJ, Chang JS. Two‐month breathing‐based walking improves anxiety, depression, dyspnoea and quality of life in chronic obstructive pulmonary disease: A randomised controlled study. Journal of clinical nursing. 2019 Oct;28(19-20):3632-40. [↑](#endnote-ref-67)
68. Wei-Li Gu, Zhen-Yu Liang, Chuan-Bing Zhu, Rong-Chang Chen. Clinical outcome of a novel breathing training manoeuvre in stable COPD patients. International Journal of Clinical and Experimental Medicine. 2018;11(9):9802-9810 [↑](#endnote-ref-68)
69. Li P, Liu J, Lu Y, Liu X, Wang Z, Wu W. Effects of long-term home-based Liuzijue exercise combined with clinical guidance in elderly patients with chronic obstructive pulmonary disease. Clinical interventions in aging. 2018 Aug 3:1391-9. [↑](#endnote-ref-69)
70. Thokchom SK, Gulati K, Ray A, Menon BK. Effects of yogic intervention on pulmonary functions and health status in patients of COPD and the possible mechanisms. Complementary Therapies in Clinical Practice. 2018 Nov 1;33:20-6. [↑](#endnote-ref-70)
71. Ranjita R, Badhai S, Hankey A, Nagendra HR. A randomized controlled study on assessment of health status, depression, and anxiety in coal miners with chronic obstructive pulmonary disease following yoga training. International Journal of Yoga. 2016 Jul;9(2):137. [↑](#endnote-ref-71)
72. Wu W, Liu X, Li P, Li N, Wang Z. Effect of Liuzijue exercise combined with elastic band resistance exercise on patients with COPD: a randomized controlled trial. Evidence-Based Complementary and Alternative Medicine. 2018 Jun 11;2018. [↑](#endnote-ref-72)
73. Liu YQ, Yan LX, Zhang LY, Song QH, Xu RM. Conspicuous effect on treatment of mild-to-moderate COPD by combining deep-breathing exercise with oxygen inhalation. International journal of clinical and experimental medicine. 2015;8(6):9918. [↑](#endnote-ref-73)
74. Xiao CM, Zhuang YC. Efficacy of Liuzijue Qigong in individuals with chronic obstructive pulmonary disease in remission. Journal of the American Geriatrics Society. 2015 Jul;63(7):1420-5. [↑](#endnote-ref-74)
75. Leelarungrayub J, Puntumetakul R, Sriboonreung T, Pothasak Y, Klaphajone J. Preliminary study: comparative effects of lung volume therapy between slow and fast deep-breathing techniques on pulmonary function, respiratory muscle strength, oxidative stress, cytokines, 6-minute walking distance, and quality of life in persons with COPD. International journal of chronic obstructive pulmonary disease. 2018 Dec 5:3909-21. [↑](#endnote-ref-75)
76. Sakhaei S, Sadagheyani HE, Zinalpoor S, Markani AK, Motaarefi H. The impact of pursed-lips breathing maneuver on cardiac, respiratory, and oxygenation parameters in COPD patients. Open access Macedonian journal of medical sciences. 2018 Oct 10;6(10):1851. [↑](#endnote-ref-76)
77. Kzar MH, Hadi AH. Effect of an inspiratory muscle strength training on dyspnoea arising from unsupported arm elevation in patients with Chronic Obstructive Pulmonary Disease. Человек. Спорт. Медицина. 2017;17(2):70-80. [↑](#endnote-ref-77)
78. Leelarungrayub J, Pinkaew D, Puntumetakul R, Klaphajone J. Effects of a simple prototype respiratory muscle trainer on respiratory muscle strength, quality of life and dyspnea, and oxidative stress in COPD patients: a preliminary study. International journal of chronic obstructive pulmonary disease. 2017 May 12:1415-25. [↑](#endnote-ref-78)
79. Heydari A, Farzad M, Ahmadi hosseini SH. Comparing inspiratory resistive muscle training with incentive spirometry on rehabilitation of COPD patients. Rehabilitation Nursing. 2015 Jul;40(4):243-8. [↑](#endnote-ref-79)
80. Fukuoka A, Ueda M, Ariyama Y, Iwai K, Kai Y, Kunimatsu M, Yoshikawa M, Uyama H, Tomoda K, Kimura H. Effect of Laughter Yoga on Pulmonary Rehabilitation in Patients with Chronic Obstructive Pulmonary Disease. Journal of Nara Medical Association, 2016;67(1,2,3):11-20. [↑](#endnote-ref-80)
81. Bhandari L, Dutta S. Health infrastructure in rural India. India infrastructure report. 2007;2007:265-85. [↑](#endnote-ref-81)
82. Mishra A. The role of the Accredited Social Health Activists in effective health care delivery: evidence from a study in South Orissa. *BMC Proceedings*; 2012: BioMed Central. [↑](#endnote-ref-82)
83. Srivastava A, Shrivastava A. ASHAs in India–The Unsung Corona Warriors. Journal of Organizational Behavior Education. 2022:37-46. [↑](#endnote-ref-83)
84. Karol GS, Pattanaik B. Community health workers and reproductive and child health care: an evaluative study on knowledge and motivation of ASHA (Accredited social health activist) Workers in Rajasthan, India. Int J Humanit Soc Sci. 2014;4(9):137-50. [↑](#endnote-ref-84)
85. Bajpai N, Dholakia RH. Improving the performance of accredited social health activists in India. Mumbai: Columbia Global Centres South Asia. 2011. [↑](#endnote-ref-85)
86. Paul PL, Pandey S. Factors influencing institutional delivery and the role of accredited social health activist (ASHA): a secondary analysis of India human development survey 2012. BMC Pregnancy and Childbirth. 2020;20(1):1-9. [↑](#endnote-ref-86)
87. Holt K, Uttekar BV, Reed R, Adams M, Kanchan L, Langer A, et al. Understanding quality of contraceptive services from women’s perspectives in Gujarat, India: a focus group study. BMJ open. 2021;11(10):e049260. [↑](#endnote-ref-87)
88. Singh AR, Pakhare A, Kokane AM, Shewade HD, Chauhan A, Singh A, et al. ‘Before reaching the last mile’-Knowledge, attitude, practice and perceived barriers related to tuberculosis directly observed therapy among ASHA workers in Central India: A mixed method study. Journal of epidemiology and global health. 2017;7(4):219-25. [↑](#endnote-ref-88)
89. Jain RB, Kumar A, Sharma E, Goel PK. Barriers to childhood vaccination as perceived by accredited social health activists and multipurpose health workers in Mewat, Haryana (India): A qualitative research. Journal of Family Medicine and Primary Care. 2020;9(8):4134. [↑](#endnote-ref-89)
90. Bhatt BR. ASHAs in rural India, the ray of hope for diabetes care. Journal of Social Health and Diabetes. 2014;2(01):018-24. [↑](#endnote-ref-90)
91. Scott K, Shanker S. Tying their hands? Institutional obstacles to the success of the ASHA community health worker programme in rural north India. AIDS care. 2010;22(sup2):1606-12. [↑](#endnote-ref-91)
92. Ramani S, Rao KD, Ryan M, Vujicic M, Berman P. For more than love or money: attitudes of student and in-service health workers towards rural service in India. Human resources for health. 2013;11:1-12. [↑](#endnote-ref-92)
93. Lall D, Engel N, Devadasan N, Horstman K, Criel B. Challenges in primary care for diabetes and hypertension: an observational study of the Kolar district in rural India. BMC health services research. 2019;19:1-11. [↑](#endnote-ref-93)
94. Kanungo S, Bhowmik K, Mahapatra T, Mahapatra S, Bhadra UK, Sarkar K. Perceived morbidity, healthcare-seeking behavior and their determinants in a poor-resource setting: observation from India. PLoS one. 2015;10(5):e0125865. [↑](#endnote-ref-94)
95. Starfield B, Shi L, Macinko J. Contribution of primary care to health systems and health. The milbank quarterly. 2005;83(3):457-502. [↑](#endnote-ref-95)
96. Srinidhi V, Karachiwala B, Iyer A, Reddy B, Mathrani V, Madhiwalla N, et al. ASHA Kirana: when digital technology empowered front-line health workers. BMJ Global Health. 2021;6(Suppl 5):e005039. [↑](#endnote-ref-96)
97. Bansal P. Blended learning in Indian higher education: Challenges and strategies. International Journal of Applied Research and Studies. 2014;3(2):1-13. [↑](#endnote-ref-97)
98. Joshi R, Alim M, Kengne AP, Jan S, Maulik PK, Peiris D, et al. Task shifting for non-communicable disease management in low and middle income countries–a systematic review. PloS one. 2014;9(8):e103754. [↑](#endnote-ref-98)
99. Shah QN, Dave PA, Loh DA, Appasani RK, Katz CL. Knowledge of and attitudes towards mental illness among ASHA and Anganwadi workers in Vadodara District, Gujarat State, India. Psychiatric Quarterly. 2019;90:303-9. [↑](#endnote-ref-99)
100. Srivastava AI, Hasan A. Bridging the Skill Gap in India: Challenges and Solutions. JIMS8M: The Journal of Indian Management & Strategy. 2016;21(1):45-54. [↑](#endnote-ref-100)
101. Kidd M. The contribution of family medicine to improving health systems: a guidebook from the World Organization of Family Doctors: CRC Press; 2020. [↑](#endnote-ref-101)
102. Hunter BM, Bisht R, Chakravarthi I, Murray SF. Demand-side financing and promotion of maternal health: what has India learnt? Economic and Political Weekly. 2014:66-73. [↑](#endnote-ref-102)
103. Husain Z. Health of the national rural health mission. Economic and Political Weekly. 2011:53-60. [↑](#endnote-ref-103)
104. Kaysin SA. The Impact of the Accredited Social Health Activists (ASHA) Program in India on the Utilization of Maternity Services from the Antepartum to the Postpartum Period: The University of North Carolina at Chapel Hill; 2017. [↑](#endnote-ref-104)
